# Supplementary material for: Clinical manifestations and health outcomes associated with Zika virus infections in adults: A systematic review
Source: PLoS Negl Trop Dis. 2021 Jul 12;15(7):e0009516. doi: 10.1371/journal.pntd.0009516 (PMC8297931; doi:10.1371/journal.pntd.0009516)
Supplement: S1 Table — Included in this supplement is a table categorizing each study by study type (‘case-control,’ ‘case series,’ ‘cross-sectional,’ or ‘cohort’) and further sub-categorizing each study into ‘surveillance,’ ‘public health-based,’ or ‘other’ (hospital-based, single-center). CDC = Centre for Disease Control, GBS = Guillain-Barré Syndrome, ICU = intensive care unit, INS = Instituto Nacional de Salud (in Colombia), US = United States WHO = World Health Organization (DOCX) [file pntd.0009516.s002.docx]

**S1 Table. Study-Type Classifications** Details of Study-Type Classifications: Surveillance or Public Health-Based Versus Other (Hospital-Based, Single-Center)

| **Type of Study** | **Author, Year** | **Public Health and/or Surveillance?** | **Details** |
| --- | --- | --- | --- |
| **Case-Control** | Anaya, 2017 | Yes | National population-based surveillance system |
|  | Cao-Lormeau, 2016 | No | Hospital-based, GBS |
|  | Geurts vanKessel, 2018 | No | Hospital-based: Dhaka Medical College and Hospital |
|  | Salinas, 2017 | Yes | Surveillance data: INS and local |
|  | Styczynski, 2017 | Yes | Reported to the Center for Information and Epidemiologic Surveillance of Bahia - passive surveillance |
|  | Gongora-Rivera, 2020 | No | Hospital-based, three reference hospitals |
|  | Rivera-Correa, 2019 | No | Hospital-based, two reference hospitals |
|  | Kozak, 2020 | No | Healthcare providers in Ontario |
| **Case Series** | Acevedo, 2017 | No | Hospital-based review |
|  | Arias, 2017 | No | Admitted to ICU (hospital-based) |
|  | Baskar, 2018 | No | Tertiary-care center in India |
|  | Chang, 2018 | No | Several departments in North Colombia |
|  | Dirlikov, 2018 | Yes | GBS surveillance system |
|  | Duijster, 2016 | Yes | Travelers returning from Surinam and the Dominican Republic to the Netherlands; declaration to share data by the WHO |
|  | Lynch, 2019 | No | Adults with ZIKV or ZIKV/GBS referred from two departments in Colombia |
|  | Sebastián, 2017 | No | Multi-center (recruited from ICU’s) |
|  | Uncini, 2018 | No | Different health-care centers in Cúcuta |
|  | Van Dyne, 2019 | Yes | Reported to Puerto Rico Department of Health (PRDH) |
|  | Watrin, 2016 | Yes | Department of Neurology Tahiti's hospital; surveillance |
|  | Chaumont, 2020 | No | Hospital-based, single-centre |
|  | Lannuzel, 2019 | No | Hospital-based, two university hospitals |
| **Cohort** | Calvet, 2018 | No | Single-center, laboratory studying febrile illnesses |
|  | da Silva, 2017 | No | Tertiary hospital |
|  | de Laval, 2018 | No | Invited military personnel and others with dermatologic manifestation in Cayenne/Korou |
|  | Kam, 2017 | No | Based on hospitalization in Campinas region of Brazil |
|  | Lozier, 2018 | Yes | Passive Arboviral Diseases Surveillance System (PADSS) |
|  | Meltzer, 2019 | Yes | Surveillance |
|  | Ng, 2018 | Yes | Teaching hospital; active case surveillance |
|  | Sokal, 2016 | No | Hospital-based |
|  | Romero Vega, 2018 | Yes | Brazilian notifiable information system |
|  | Petridou, 2019 | Yes | Rare and imported pathogens library (RIPL) |
|  | Hunsberger, 2020 | No | Two primary health care centres |
|  | Crespillo-Andújar, 2019 | No | Hospital-based, single centre |
|  | El Sahly, 2018 | No | Passive referrals and active-case finding (health care facilities and laboratory-based screening) |
| **Cross-Sectional** | Adams, 2016 | Yes | Surveillance network |
|  | Armstrong, 2016 | Yes | US Department of Health and Human Services |
|  | Azeredo, 2018 | No | Healthy Unit, Campo Grande |
|  | Boggild, 2017 | Yes | CanTravNet Surveillance System |
|  | Brasil, 2016 | Yes | Surveillance clinic |
|  | Brenciaglia, 2018 | Yes | Research team but for surveillance |
|  | da Silvo Brito, 2018 | Yes | Surveillance, General Hospital of Nova Iguacu |
|  | Daudens-Vaysse, 2016 | Yes | Reporting, surveillance system |
|  | Duffy, 2009 | Yes | Hospital-based surveillance, 4 healthcare centers on Yap |
|  | Francis, 2018 | Yes | Surveillance |
|  | Hall, 2018 | Yes | National Arboviral Surveillance System |
|  | Hamer, 2017 | Yes | GeoSentinel data collection, surveillance |
|  | Ho, 2017 | Yes | Surveillance |
|  | Huits, 2019 | No | Travel clinic, Institute of Tropic Medicine, Antwerp |
|  | Jimenez Corona, 2016 | Yes | Surveillance |
|  | Journel, 2017 | Yes | US Department of Health and Human Services |
|  | Lee, 2016 | Yes | CDC Surveillance data |
|  | Malta, 2017 | Yes | Surveillance |
|  | McGibbon, 2018 | Yes | Surveillance |
|  | Méndez, 2017 | Yes | INS, Surveillance, Colombia |
|  | Millet, 2017 | Yes | Public health, Barcelona, reporting system |
|  | Parra, 2016 | No | 6 university-based centers |
|  | Rozé, 2017 | No | Hospital-based, University Hospital of Martinique (UHM) |
|  | Ryan, 2017 | Yes | Surveillance |
|  | Schirmer, 2018 | Yes | Surveillance, Veterans Health Administration (VHA) |
|  | Simon, 2018 | No | Tertiary hospital |
|  | Thomas, 2016 | Yes | CDC Surveillance |
|  | Vroon, 2017 | No | Emergency department data |
|  | Webster-Kerr, 2017 | Yes | Arboviral surveillance |
|  | Grajales-Muniz, 2019 | Yes | Mexican Social Security Institute (IMSS) |
|  | Valle, 2018 | Yes | Clinic-based, academic travel clinic |
|  | Martinez, 2019 | Yes | National Surveillance Network (RENAVE) |
|  | Silva, 2019 | Yes | Public Health Emergency Unit |
|  | Mercado-Reyes, 2018 | Yes | National Surveillance System in Public Health (SIVIGILA) |
|  | Garcell, 2019 | No | Hospital-based, single-centre |
|  | Del Carpio-Orantes, 2020 | No | Ambulatory centre |
|  | Castañeda-Martinez, 2019 | Yes | Surveillance |
|  | Sharma, 2019 | Yes | Surveillance community study, household screening |
|  | Vazquez, 2019 | Yes | Surveillance |
|  | Phan, 2019 | Yes | Surveillance |

CDC = Centre for Disease Control, GBS = Guillain-Barré Syndrome, ICU = intensive care unit, INS = Instituto Nacional de Salud (in Colombia), US = United States WHO = World Health Organization
