# Supplementary figures and images for: Clinical manifestations and health outcomes associated with Zika virus infections in adults: A systematic review
Source: PLoS Negl Trop Dis. 2021 Jul 12;15(7):e0009516. doi: 10.1371/journal.pntd.0009516 (PMC8297931; doi:10.1371/journal.pntd.0009516)

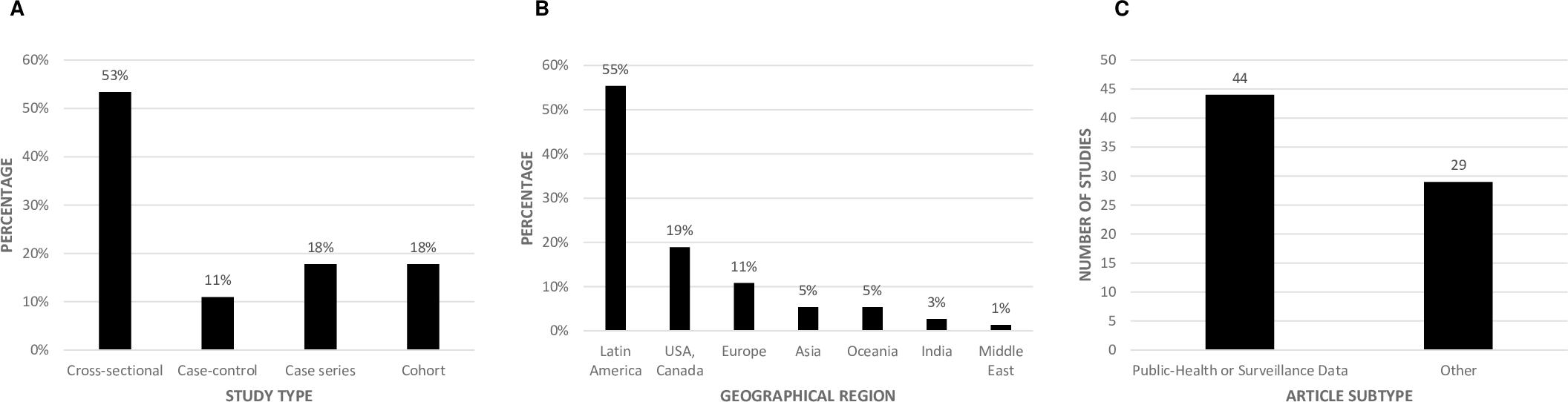

Supplement: S1 Fig — (A) Classified by study-type (classified by authors of systematic review), (B) Classified by geographic location of subjects and (C) Classified by involvement in public health reporting or surveillance versus other (purely hospital-based, healthcare center-based, population-based, travel-clinic based). (TIF) [file pntd.0009516.s003.tif]

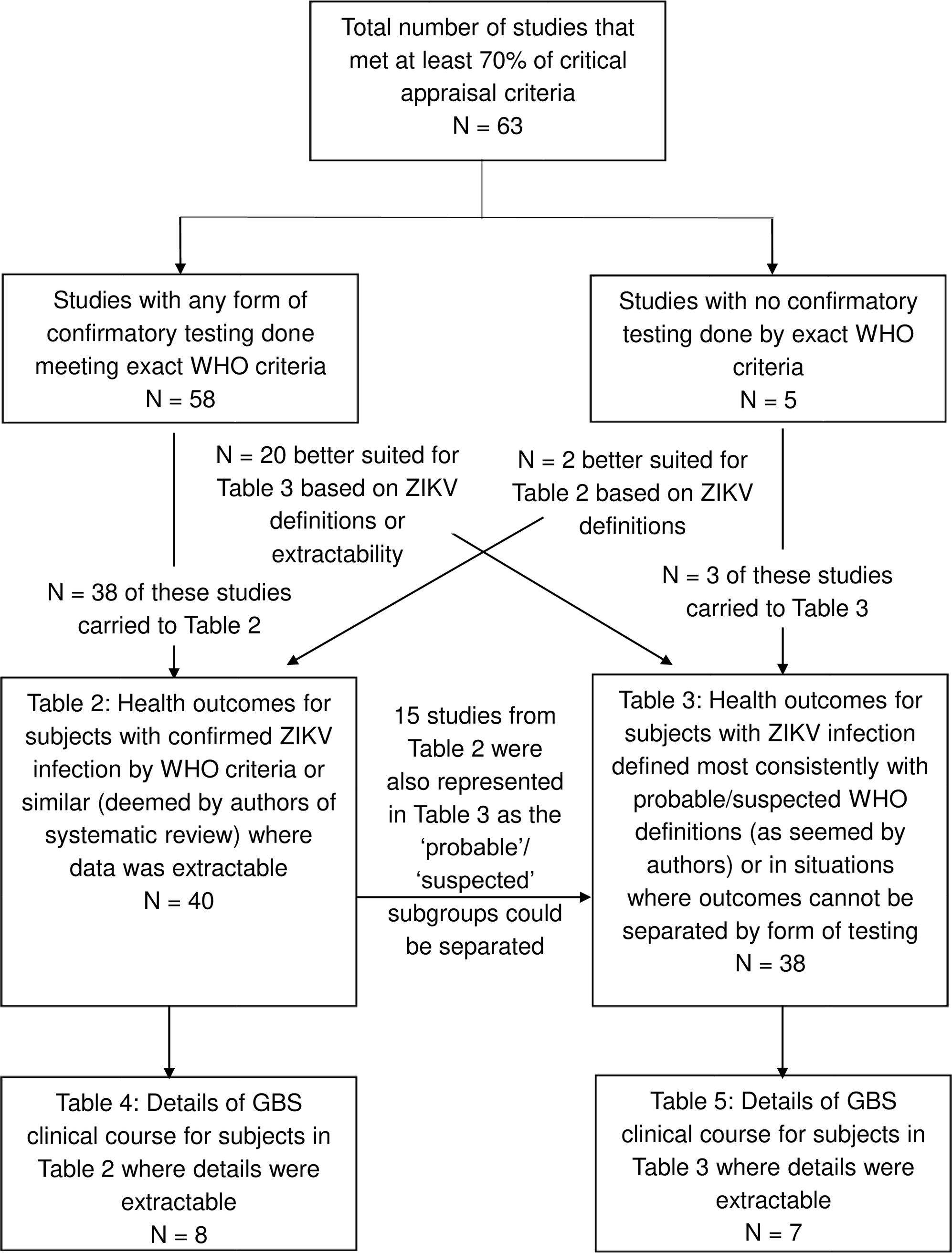

Supplement: S2 Fig — This figure depicts the grouping of primary studies into tables within our manuscript based on ZIKV testing methodologies and details included within each primary study. (TIF) [file pntd.0009516.s004.tif]
