# Supplementary material for: Clinical manifestations and health outcomes associated with Zika virus infections in adults: A systematic review
Source: PLoS Negl Trop Dis. 2021 Jul 12;15(7):e0009516. doi: 10.1371/journal.pntd.0009516 (PMC8297931; doi:10.1371/journal.pntd.0009516)
Supplement: S1 Text — Included here the search strategy and review process with details on the searches performed from five of the included databases. (DOCX) [file pntd.0009516.s005.docx]

**S1 Text. Full Search Strategy for Systematic Review**

**Search strategy**

- An information specialist with expertise in systematic reviews designed and conducted the search strategy.
- The information specialist created the initial literature search in MEDLINE Ovid and thereafter converted it into the syntax of the other databases: Embase (Ovid), PubMed, CINAHL (EBSCO), LILACS (Literatura Latino Americana em Ciências da Saúde) and WHO's ICTRP clinical trials registries database.
- At the outset, we searched databases for publications from inception until April 2018, then updated the search until 15 September 2020
- We deduplicated final results in Endnote followed by manual deduplication during the screening process.
- Full search strategies are included below.

**Review process**

- Non-English abstracts were reviewed by native speakers of the language.
- As this was one of a series of ZIKV systematic reviews involving different age groups and populations, a first round of full-text review identified publications with possible adult data.
- Exclusion criteria were applied to exclude full-text articles that were conference abstracts, letters to the editor, or had N < 10.
- Data was then extracted on a pre-designed, pilot-tested data extraction form on Microsoft Excel, with the following major sections and subsections within each of these:
  - Study type
  - Subject demographic characteristics
  - Zika virus signs and symptoms and clinical outcomes
- Discrepancies were resolved through consensus or by consulting a third reviewer
- Critical appraisal was then performed using the Joanna Briggs Institute Critical Appraisal Tools

**Full search strategy**

**Topic: Zika infection sequelae in children, adults and pregnant women**

Date: Apr 12, 2018

Update: 17 Dec 2018

Update: 16 Jan 2019

Update: 15 Sep 2020

Database(s):

Ovid MEDLINE: Epub Ahead of Print, In-Process & Other Non-Indexed Citations,

Ovid MEDLINE® Daily and Ovid MEDLINE®

Embase Classic+Embase

CINAH

PubMedU.S. National Library of Medicine

LILACS (Literatura Latino Americana em Ciências da Saúde)

**MEDLINE SEARCH**

Databases searched: Ovid MEDLINE: Epub Ahead of Print, In-Process & Other Non-Indexed Citations, Ovid MEDLINE® Daily and Ovid MEDLINE® 1946-Present

Search Strategy name: Zika virus minus pub types MEDLINE

**MEDLINE Search Strategy**

| **#** | **Searches** |
| --- | --- |
| 1 | Zika Virus/ or Zika Virus Infection/ or (Zika or ZikV).mp,kf. |
| 2 | ("clinical conference" or comment or congresses or "conference abstract" or dissertation or editorial or festschrift or "historical article" or letter or news or "newspaper article" or summary or addresses or biography or "case reports" or directory or interview or lectures or "legal cases" or legislation or "patient education handout" or "popular works" or "consensus development conference" or " consensus development conference, nih" or " practice guideline").pt. |
| 3 | 1 not 2 |

**EMBASE SEARCH**

**Databases searched:** **Embase Classic+Embase**1947 to 2020 September 15

**Search Strategy name:** Zika virus minus pub types EMBASE

**Embase Search Strategy**

| **#** | **Searches** |
| --- | --- |
| 1 | exp Zika virus/ or exp Zika fever/ or exp Zika virus vaccine/ or (Zika or ZikV).mp,hw. |
| 2 | (comment or editorial or historical article or letter or biography or festschrift or interview? or lecture? or "legal case?" or "patient education handout" or congresses).pt. |
| 3 | 1 not 2 |

**CINHAL SEARCH**

**Databases searched:**

Interface - EBSCOhost Research Databases;

Database – CINAHL

**CINHAL Search Strategy**

| [**Search ID#**](javascript:__doPostBack('ctl00$ctl00$FindField$FindField$historyControl$ReorderHistoryLink','')) | **Search Terms** | **Search Options** |
| --- | --- | --- |
| S5 | S4 not S3 | **Search modes** - Boolean/Phrase |
| S4 | S1 OR S2 | **Search modes** - Boolean/Phrase |
| S3 | S1 OR S2 | **Limiters** - Publication Type: Abstract, Algorithm, Anecdote, Bibliography, Biography, Book, Book Chapter, Book Review, Brief Item, Cartoon, CEU, Code of Ethics, Commentary, Computer Program, Consumer/Patient Teaching Materials, Directories, Editorial, Equations & Formulas, Exam Questions, Forms, Games, Glossary, Historical Material, Individual Testimonial Website, Interview  **Search modes** - Boolean/Phrase |
| S2 | TX Zika OR TX ZikV | **Search modes** - Boolean/Phrase |
| S1 | (MH "Zika Virus") OR (MH "Zika Virus Infections") | **Search modes** - Boolean/Phrase |

**PUBMED SEARCH**

**Databases searched:** PubMed, [U.S. National Library of Medicine](https://www.nlm.nih.gov/)

**PubMed Search Strategy**

| **#** | **Searches** |
| --- | --- |
| 1 | (((((("Zika Virus"[Mesh]) OR "Zika Virus Infection"[Mesh]))) OR ((Zika) OR ZikV))) NOT (((((("Zika Virus"[Mesh]) OR "Zika Virus Infection"[Mesh]))) OR ((Zika) OR ZikV)) AND ( ( Addresses[ptyp] OR Autobiography[ptyp] OR Bibliography[ptyp] OR Biography[ptyp] OR pubmed books[filter] OR Comment[sb] OR Congresses[ptyp] OR Consensus Development Conference[ptyp] OR Consensus Development Conference, NIH[ptyp] OR Dictionary[ptyp] OR Directory[ptyp] OR Editorial[ptyp] OR Festschrift[ptyp] OR Guideline[ptyp] OR Historical Article[ptyp] OR Interactive Tutorial[ptyp] OR Interview[ptyp] OR Lectures[ptyp] OR Legal Cases[ptyp] OR Legislation[ptyp] OR Letter[ptyp] OR News[ptyp] OR Newspaper Article[ptyp] OR Overall[ptyp] OR Patient Education Handout[ptyp] OR Periodical Index[ptyp] OR Portraits[ptyp] OR Practice Guideline[ptyp] OR Research Support, American Recovery and Reinvestment Act[ptyp] OR Research Support, N I H, Extramural[ptyp] OR Research Support, N I H, Intramural[ptyp] OR Research Support, Non U S Gov't[ptyp] OR Research Support, U S Gov't, Non P H S[ptyp] OR Research Support, U S Gov't, P H S[ptyp] OR Research Support, U.S. Government[ptyp] OR Retracted Publication[sb] OR Retraction of Publication[sb] OR Scientific Integrity Review[ptyp] OR Technical Report[ptyp] OR Video-Audio Media[ptyp] OR Webcasts[ptyp]))) |

**LILACS SEARCH**

**Databases searched:** LILAC

Search Strategy:

 (tw:(tw:(zika OR zikv) OR (mh:("Zika Virus Infection")) OR (mh:("Zika Virus"))))
