# Supplementary material for: Clinical manifestations and health outcomes associated with Zika virus infections in adults: A systematic review
Source: PLoS Negl Trop Dis. 2021 Jul 12;15(7):e0009516. doi: 10.1371/journal.pntd.0009516 (PMC8297931; doi:10.1371/journal.pntd.0009516)
Supplement: S2 Text — Protocol amendment has been submitted to include three authors on the protocol. (PDF) [file pntd.0009516.s006.pdf]

NHS
National Institute for Health Research

UNIVERSITY of York
Centre for Reviews and Dissemination

Systematic review

1. * Review title.

Give the title of the review in English

Clinical manifestations and health outcomes associated with zika virus infections in adults. A systematic review

2. Original language title.

For reviews in languages other than English, give the title in the original language. This will be displayed with the English language title.

3. * Anticipated or actual start date.

Give the date the systematic review started or is expected to start.

01/05/2018

4. * Anticipated completion date.

Give the date by which the review is expected to be completed.

30/09/2018

5. * Stage of review at time of this submission.

Tick the boxes to show which review tasks have been started and which have been completed. Update this field each time any amendments are made to a published record.

Reviews that have started data extraction (at the time of initial submission) are not eligible for inclusion in PROSPERO. If there is later evidence that incorrect status and/or completion date has been supplied, the published PROSPERO record will be marked as retracted.

This field uses answers to initial screening questions. It cannot be edited until after registration.

The review has not yet started: No

Review stage	Started	Completed
Preliminary searches	Yes	Yes
Piloting of the study selection process	Yes	Yes
Formal screening of search results against eligibility criteria	Yes	Yes
Data extraction	Yes	Yes
Risk of bias (quality) assessment	Yes	Yes
Data analysis	Yes	Yes

Provide any other relevant information about the stage of the review here.

6. * Named contact.

The named contact is the guarantor for the accuracy of the information in the register record. This may be any member of the review team.

Beate Sander

Email salutation (e.g. "Dr Smith" or "Joanne") for correspondence:

Dr. Sander

7. * Named contact email.

Give the electronic email address of the named contact.

Beate.Sander@uhnresearch.ca

8. Named contact address

Give the full institutional/organisational postal address for the named contact.

University Health Network

Toronto General Hospital, Eaton Building, 10th Floor, Room 248

200 Elizabeth Street, Toronto, ON

M5G 2C4

9. Named contact phone number.

Give the telephone number for the named contact, including international dialling code.

7789608738416-634-8020

10. * Organisational affiliation of the review.

Full title of the organisational affiliations for this review and website address if available. This field may be completed as 'None' if the review is not affiliated to any organisation.

Toronto Health Economics and Technology Assessment (THETA) Collaborative

University Health Network

University of Toronto

Hospital for Sick Children

Mount Sinai Hospital

Organisation web address:

http://theta.utoronto.ca/HomePage/Home

http://www.uhn.ca/

https://www.utoronto.ca/

https://www.sickkids.ca/

https://www.mountsinai.on.ca/

11. * Review team members and their organisational affiliations.

Give the personal details and the organisational affiliations of each member of the review team. Affiliation refers to groups or organisations to which review team members belong. **NOTE: email and country now MUST be entered for each person, unless you are amending a published record.**

Miss Panashe Ethaniel Tombindo. University of Toronto
Miss Sheliza Halani. University of Toronto
Mr Ryan O'Reilly. University of Toronto
Ms Joanna M. Bielecki. Toronto Health Economics and Technology Assessment (THETA) Collaborative
Mr Justin Boyle. University of Toronto
Dr Laura Erdman. Division of Infectious Diseases, Hospital for Sick Children, Toronto
Dr Shaun Morris. Division of Infectious Diseases, Hospital for Sick Children, Toronto
Dr Kellie Murphy. Mt Sinai Hospital, Toronto
Mr Rafael Neves Miranda. Toronto Health Economics and Technology Assessment (THETA) Collaborative
Ms Lauren Ramsay. University of Toronto
Dr Clare Whitehead. University of Toronto
Mr Raphael Ximenes. Toronto Health Economics and Technology Assessment (THETA) Collaborative
Dr Beate Sander. Toronto Health Economics and Technology Assessment Collaborative; University of Toronto
Mr Carsten Krueger. Hospital for Sick Children, Toronto
Ms Shannon Willmott. The Hospital for Sick Children, Toronto

12. * Funding sources/sponsors.

Details of the individuals, organizations, groups, companies or other legal entities who have funded or sponsored the review.

Canadian Institutes of Health Research (CIHR), ZV3 149784

Grant number(s)

State the funder, grant or award number and the date of award

13. * Conflicts of interest.

List actual or perceived conflicts of interest (financial or academic).

None

14. Collaborators.

Give the name and affiliation of any individuals or organisations who are working on the review but who are not listed as review team members. **NOTE: email and country must be completed for each person, unless you are amending a published record.**

15. * Review question.

State the review question(s) clearly and precisely. It may be appropriate to break very broad questions down into a series of related more specific questions. Questions may be framed or refined using PI(E)COS or similar where relevant.

What are the clinical manifestations and health outcomes associated with Zika virus infection in adults?

16. * Searches.

State the sources that will be searched (e.g. Medline). Give the search dates, and any restrictions (e.g. language or publication date). Do NOT enter the full search strategy (it may be provided as a link or attachment below.)

We will conduct a systematic review following the PRISMA (Preferred Reporting Items for Systematic Reviews and Meta-Analyses) guidelines. Eligible studies will be identified through a systematic comprehensive search of the following databases: MEDLINE (Ovid), Embase (Ovid), PubMed, CINAHL (EBSCO), LILACS (Literatura Latino Americana em Ciências da Saúde) and WHO's ICTRP clinical trials registries database. PROSPERO register will also be searched for all active or completed systematic reviews.

The search strategy was designed and conducted by an information specialist experienced in systematic reviews (JB) following the Cochrane systematic review methodology. The initial search will be designed in MEDLINE Ovid and subsequently translated into other databases' syntax. It includes controlled vocabulary (MeSH) and natural language terms in the following concept areas: Zika virus and Zika infection. There will be no restrictions in terms of language or publication period, however, we will exclude publications such as editorials, letters and news articles. Electronic database searches will be supplemented with: inspection of reference lists of relevant articles, and hand searching of pertinent journals.

We will also perform a search of the grey literature to identify reports from governmental and non-

governmental organizations and pending research using resources such as the Grey Literature Report publication of The New York Academy of Medicine, the Grey Matters: a practical tool for searching health-related grey literature checklist produced by the Canadian Agency for Drugs and Technologies in Health (CADTH). We will also search targeted websites, e.g, PAHO, CDC. We may consult with experts to ensure all key sources have been identified.

17. URL to search strategy.

Upload a file with your search strategy, or an example of a search strategy for a specific database, (including the keywords) in pdf or word format. In doing so you are consenting to the file being made publicly accessible. Or provide a URL or link to the strategy. Do NOT provide links to your search **results**.

Alternatively, upload your search strategy to CRD in pdf format. Please note that by doing so you are consenting to the file being made publicly accessible.

Do not make this file publicly available until the review is complete

18. * Condition or domain being studied.

Give a short description of the disease, condition or healthcare domain being studied in your systematic review.

Zika virus is an enveloped single-stranded RNA virus in the Flavivirus family. Transmission occurs via the bite of an infected Aedes mosquito, or sexual or vertical transmission. Zika virus was first isolated in Uganda in 1947 and over the next decades sporadic cases were documented in Africa and Asia. Clinical manifestations range from asymptomatic infection to a self-limiting febrile illness with rash and arthralgia, to neurological complications such as Guillain-Barre Syndrome and encephalitis.

In the mid-2010s, a Zika virus epidemic emerged in Central and South America and the Caribbean. During this time, the teratogenic effects of congenital exposure to Zika virus were recognized -- particularly brain malformations and other neuro-ophthalmologic abnormalities, likely related to viral neurotropism.

Zika virus infection requires further study to characterize the acute clinical manifestations and short-and long-term health outcomes. Results of this study will help guide clinical care, inform models to assess interventions, and identify areas for future research.

19. * Participants/population.

Specify the participants or populations being studied in the review. The preferred format includes details of both inclusion and exclusion criteria.

Adults - defined as men and women above the age of 18 years, who have been infected with the Zika virus.

This population will include pregnant women

20. * Intervention(s), exposure(s).

Give full and clear descriptions or definitions of the interventions or the exposures to be reviewed. The preferred format includes details of both inclusion and exclusion criteria.

Adults diagnosed with Zika virus infections. We will include all methods of diagnosis, with subgroup analyses

performed to assess potential differences between confirmed or probable cases using standardized (e.g., those published by PAHO, WHO, and CDC) or other case definitions.

21. * Comparator(s)/control.

Where relevant, give details of the alternatives against which the intervention/exposure will be compared (e.g. another intervention or a non-exposed control group). The preferred format includes details of both inclusion and exclusion criteria.

Uninfected healthy adults without evidence of Zika virus infection. Studies investigating clinical manifestations and outcomes without controls will also be included given the limited nature of existing literature on Zika virus infection

22. * Types of study to be included.

Give details of the study designs (e.g. RCT) that are eligible for inclusion in the review. The preferred format includes both inclusion and exclusion criteria. If there are no restrictions on the types of study, this should be stated.

We will include primary observational studies (cross-sectional, case-control and cohort studies) to assess the clinical manifestations and health outcomes of Zika virus infections. Given the relative paucity of data on Zika virus infection and potential outcomes, we will include case series and case reports, with attention to the risk of bias. Randomized controlled trials (RCT) that investigate the outcomes of interest will also be included; those that focus on treatment safety and efficacy will be excluded from the review. We will exclude abstracts and conference proceedings as it is difficult to assess methodology and data quality. Studies in animals will also be excluded.

23. Context.

Give summary details of the setting or other relevant characteristics, which help define the inclusion or exclusion criteria.

All geographic settings will be included.

24. * Main outcome(s).

Give the pre-specified main (most important) outcomes of the review, including details of how the outcome is defined and measured and when these measurement are made, if these are part of the review inclusion criteria.

Frequency of clinical manifestations and health outcomes (e.g., Guillain-Barre Syndrome, mortality).

Measures of effect

Please specify the effect measure(s) for you main outcome(s) e.g. relative risks, odds ratios, risk difference, and/or 'number needed to treat.

Mean, Correlation

25. * Additional outcome(s).

List the pre-specified additional outcomes of the review, with a similar level of detail to that required for main outcomes. Where there are no additional outcomes please state ‘None’ or ‘Not applicable’ as appropriate to the review

Prognostic factors, duration of illness, markers of severity

Measures of effect

Please specify the effect measure(s) for you additional outcome(s) e.g. relative risks, odds ratios, risk difference, and/or 'number needed to treat.

Mean, Correlation

26. * Data extraction (selection and coding).

Describe how studies will be selected for inclusion. State what data will be extracted or obtained. State how this will be done and recorded.

Two reviewers will independently screen titles and abstracts of identified records, followed by reviewing the full text against inclusion/exclusion criteria. Discrepancies will be resolved through consensus or consultation with a third reviewer or the larger research team. We will extract data on a pre-designed, pilot-tested data extraction form including study characteristics, patient characteristics and results on descriptive statistics and measures of association (frequency of clinical manifestations and sequelae, timing and method of diagnosis). Discrepancy in data extraction will be resolved through consensus or consultation with a third reviewer or the larger research team

27. * Risk of bias (quality) assessment.

State which characteristics of the studies will be assessed and/or any formal risk of bias/quality assessment tools that will be used.

Two reviewers will independently assess the quality of eligible primary studies through critical appraisals specific to the study design, and resolve disagreements through consensus or consultation with a third reviewer or the larger research team. Studies will be assessed using the Joanna Briggs Institute (JBI) Critical Appraisal tools.

We will assess the risk of bias of all eligible studies, but will not exclude studies from the final analysis based on perceived strength or quality of evidence. Instead, studies will be stratified by risk of bias in sensitivity analyses. We will pilot the tools on a preliminary set of studies to ensure consistency and accuracy in assessment.

28. * Strategy for data synthesis.

Describe the methods you plan to use to synthesise data. This **must not be generic text** but should be **specific to your review** and describe how the proposed approach will be applied to your data. If meta-analysis is planned, describe the models to be used, methods to explore statistical heterogeneity, and software package to be used.

We will provide a qualitative and quantitative synthesis of the findings from the included studies where appropriate depending on the heterogeneity of the included studies. If studies are sufficiently homogeneous in terms of design and comparator, we will conduct an aggregate data meta-analysis using a random-effects model.

29. * Analysis of subgroups or subsets.

State any planned investigation of ‘subgroups’. Be clear and specific about which type of study or participant will be included in each group or covariate investigated. State the planned analytic approach.

We will explore whether outcomes vary with age at infection, sex, pregnancy status, immune status, certainty of Zika infection diagnosis (i.e., confirmed, probable, clinical, etc), geographic region, study type, or risk of bias. These analyses will not be pre-specified as the approach will be guided by the data collected.

30. * Type and method of review.

Select the type of review, review method and health area from the lists below.

Type of review

Cost effectiveness
No

Diagnostic
No

Epidemiologic
No

Individual patient data (IPD) meta-analysis
No

Intervention
No

Living systematic review
No

Meta-analysis
No

Methodology
No

Narrative synthesis
No

Network meta-analysis
No

Pre-clinical
No

Prevention
No

Prognostic
No

Prospective meta-analysis (PMA)
No

Review of reviews
No

Service delivery
No

Synthesis of qualitative studies
No

Systematic review
Yes

Other
No

Health area of the review

Alcohol/substance misuse/abuse
No

Blood and immune system
No

Cancer
No

Cardiovascular
No

Care of the elderly
No

Child health
No

Complementary therapies
No

COVID-19
No

Crime and justice
No

Dental
No

Digestive system
No

Ear, nose and throat
No

Education
No

Endocrine and metabolic disorders
No

Eye disorders
No

General interest
No

Genetics
No

Health inequalities/health equity

No

Infections and infestations
Yes

International development
No

Mental health and behavioural conditions
No

Musculoskeletal
No

Neurological
Yes

Nursing
No

Obstetrics and gynaecology
No

Oral health
No

Palliative care
No

Perioperative care
No

Physiotherapy
No

Pregnancy and childbirth
No

Public health (including social determinants of health)
No

Rehabilitation
No

Respiratory disorders
No

Service delivery
No

Skin disorders
No

Social care
No

Surgery
No

Tropical Medicine
Yes

Urological
No

Wounds, injuries and accidents
No

Violence and abuse
No

31. Language.

Select each language individually to add it to the list below, use the bin icon to remove any added in error.

There is an English language summary.

32. * Country.

Select the country in which the review is being carried out. For multi-national collaborations select all the countries involved.

Canada

33. Other registration details.

Name any other organisation where the systematic review title or protocol is registered (e.g. Campbell, or The Joanna Briggs Institute) together with any unique identification number assigned by them. If extracted data will be stored and made available through a repository such as the Systematic Review Data Repository (SRDR), details and a link should be included here. If none, leave blank.

34. Reference and/or URL for published protocol.

If the protocol for this review is published provide details (authors, title and journal details, preferably in Vancouver format)

Add web link to the published protocol.

Or, upload your published protocol here in pdf format. Note that the upload will be publicly accessible.

No I do not make this file publicly available until the review is complete

Please note that the information required in the PROSPERO registration form must be completed in full even if access to a protocol is given.

35. Dissemination plans.

Do you intend to publish the review on completion?

Yes

Give brief details of plans for communicating review findings.?

Results of the systematic review will be published in peer reviewed journals.

36. Keywords.

Give words or phrases that best describe the review. Separate keywords with a semicolon or new line. Keywords help PROSPERO users find your review (keywords do not appear in the public record but are included in searches). Be as specific and precise as possible. Avoid acronyms and abbreviations unless these are in wide use.

Zika, Zika virus, Zika infection, clinical manifestations, health outcomes

37. Details of any existing review of the same topic by the same authors.

If you are registering an update of an existing review give details of the earlier versions and include a full bibliographic reference, if available.

38. * Current review status.

Update review status when the review is completed and when it is published.New registrations must be ongoing so this field is not editable for initial submission.
Please provide anticipated publication date

Review_Ongoing

39. Any additional information.

Provide any other information relevant to the registration of this review.

40. Details of final report/publication(s) or preprints if available.

Leave empty until publication details are available OR you have a link to a preprint (NOTE: this field is not editable for initial submission). List authors, title and journal details preferably in Vancouver format.

Give the link to the published review or preprint.
