# Supplementary material for: Clinical manifestations and health outcomes associated with Zika virus infections in adults: A systematic review
Source: PLoS Negl Trop Dis. 2021 Jul 12;15(7):e0009516. doi: 10.1371/journal.pntd.0009516 (PMC8297931; doi:10.1371/journal.pntd.0009516)
Supplement: S3 Text — S3A. Table JBI Critical Appraisal Tool Questionnaire for Case-Control Studies Applied to ZIKV Systematic Review. S3B Table. JBI Critical Appraisal Tool Questionnaire for Case Series Applied to ZIKV Systematic Review. S3C Table. JBI Critical Appraisal Tool Questionnaire for Cohort Studies Applied to ZIKV Systematic Review. S3D Table. JBI Critical Appraisal Tool Questionnaire for Cross-Sectional Studies Applied to ZIKV Systematic Review. Note: Causation cannot be inferred from cross-sectional studies, though if no statistical analysis was performed, a point was subtracted from the critical appraisal for the particular study. (DOCX) [file pntd.0009516.s007.docx]

**S3 Text. Joanna Briggs Institute (JBI) Critical Appraisal Tool and ZIKV Adult Population Results

S3A Table. JBI Critical Appraisal Tool Questionnaire for Case-Control Studies Applied to ZIKV Systematic Review**

| Study | Q1 | Q2 | Q3 | Q4 | Q5 | Q6 | Q7 | Q8 | Q9 | Q10 | Total QA (%) |
| --- | --- | --- | --- | --- | --- | --- | --- | --- | --- | --- | --- |
| Anaya, 2017 | Y | Y | Y | Y | **Y** | Y | Y | Y | Y | Y | 100 |
| Cao-Lormeau, 2016 | U | Y | Y | Y | **Y** | Y | Y | Y | Y | Y | 90 |
| Geurts vanKessel, 2018 | Y | Y | Y | Y | **Y** | Y | Y | Y | Y | Y | 100 |
| Salinas, 2017 | Y | Y | N/A | Y | **Y** | Y | Y | Y | Y | Y | 90 |
| Styczynski, 2017 | Y | Y | N/A | Y | **Y** | Y | Y | Y | Y | Y | 100 |
| Gongora-Rivera, 2020 | Y | Y | Y | Y | **Y** | N | N/A | Y | Y | Y | 88.9 |
| Rivera-Correa, 2019 | Y | U | Y | Y | **Y** | Y | Y | Y | Y | Y | 90 |
| Kozak, 2020 | Y | Y | Y | Y | **Y** | Y | Y | Y | Y | Y | 100 |

Q1. Were the groups comparable other than the presence of disease in cases or the absence of disease in controls?

Q2. Were cases and controls matched appropriately?

Q3. Were the same criteria used for identification of cases and controls?

Q4. Was exposure measured in a standard, valid and reliable way?

Q5. Was exposure measured in the same way for cases and controls?

Q6. Were confounding factors identified?

Q7. Were strategies to deal with confounding factors stated?

Q8. Were outcomes assessed in a standard, valid and reliable way for cases and controls?

Q9. Was the exposure period of interest long enough to be meaningful?

Q10. Was appropriate statistical analysis used?

**S3B Table. JBI Critical Appraisal Tool Questionnaire for Case Series Applied to ZIKV Systematic Review**

| Study | Q1 | Q2 | Q3 | Q4 | Q5 | Q6 | Q7 | Q8 | Q9 | Q10 | Total QA (%) |
| --- | --- | --- | --- | --- | --- | --- | --- | --- | --- | --- | --- |
| Acevedo, 2017 | N | Y | Y | Y | U | N | Y | Y | N | N/A | 55.6 |
| Arias, 2016 | Y | Y | Y | Y | U | Y | Y | Y | Y | N | 80 |
| Baskar, 2018 | Y | Y | Y | Y | Y | Y | Y | N | N | Y | 80 |
| Chang, 2018 | Y | Y | Y | U | U | Y | Y | Y | N | Y | 70 |
| Dirlikov, 2018 | Y | Y | Y | Y | Y | N | Y | Y | Y | Y | 90 |
| Duijster, 2016 | N | Y | Y | U | U | Y | Y | Y | U | N/A | 55.6 |
| Lynch, 2018 | Y | Y | Y | N | N | Y | Y | Y | N | Y | 70 |
| Sebastián, 2017 | Y | Y | Y | U | N | Y | Y | Y | N | N/A | 66.7 |
| Uncini, 2017 | Y | Y | Y | Y | U | N | Y | Y | N | Y | 70 |
| Van Dyne, 2018 | Y | Y | Y | Y | Y | Y | Y | Y | N | N | 80 |
| Watrin, 2016 | Y | Y | Y | Y | Y | Y | Y | Y | Y | Y | 100 |
| Chaumont, 2020 | Y | Y | Y | Y | Y | Y | Y | Y | Y | Y | 100 |
| Soto-Hernandez, 2019 | Y | Y | Y | U | U | Y | Y | Y | Y | N/A | 77.8 |
| Lannuzel, 2019 | Y | Y | Y | Y | Y | Y | Y | Y | Y | Y | 100 |

Q1. Were there clear criteria for inclusion in the case series?

Q2. Was the condition measured in a standard, reliable way for all participants included in the case series?

Q3. Were valid methods used for identification of the condition for all participants included in the case series?

Q4. Did the case series have consecutive inclusion of participants?

Q5. Did the case series have complete inclusion of participants?

Q6. Was there clear reporting of the demographics of the participants in the study?

Q7. Was there clear reporting of clinical information of the participants?

Q8. Were the outcomes or follow up results of cases clearly reported?

Q9. Was there clear reporting of the presenting site(s)/clinic(s) demographic information?

Q10. Was statistical analysis appropriate?

| Study | Q1 | Q2 | Q3 | Q4 | Q5 | Q6 | Q7 | Q8 | Q9 | Q10 | Q11 | Total QA (%) |
| --- | --- | --- | --- | --- | --- | --- | --- | --- | --- | --- | --- | --- |
| Calvet, 2018 | Y | Y | Y | Y | Y | Y | Y | U | U | N/A | Y | 80 |
| da Silva, 2017 | Y | Y | Y | Y | Y | N/A | Y | Y | N | N/A | Y | 88.9 |
| de Laval, 2018 | N/A | N/A | Y | Y | Y | Y | Y | Y | Y | N | Y | 88.9 |
| Kam, 2017 | N | N/A | Y | N | N/A | N/A | Y | Y | U | N/A | Y | 57.1 |
| Lozier, 2017 | Y | Y | Y | Y | Y | U | Y | Y | U | N/A | Y | 80 |
| Meltzer, 2019 | Y | Y | Y | Y | Y | Y | Y | Y | U | N/A | Y | 90 |
| Ng, 2018 | N/A | N/A | Y | Y | Y | Y | Y | Y | Y | N/A | Y | 100 |
| Sokal, 2016 | N/A | N/A | Y | N | N/A | N | Y | Y | Y | N/A | N/A | 66.7 |
| Romero Vega, 2018 | NA | NA | Y | Y | Y | Y | Y | Y | Y | NA | Y | 100 |
| Petridou, 2019 | NA | NA | Y | Y | Y | Y | Y | U | N | NA | NA | 71.4 |
| Hunsberger, 2020 | NA | NA | Y | Y | Y | N | Y | Y | Y | Y | Y | 88.9 |
| Crespillo-Andújar, 2020 | Y | Y | Y | Y | Y | N | Y | Y | U | NA | Y | 80 |
| El Sahly, 2018 | Y | Y | Y | Y | Y | N | Y | Y | Y | NA | Y | 90 |

**S3C. JBI C Table ritical Appraisal Tool Questionnaire for Cohort Studies Applied to ZIKV Systematic Review**

Q1. Were the two groups similar and recruited from the same population?

Q2. Were the exposures measured similarly to assign people to both exposed and unexposed groups?

Q3. Was the exposure measured in a valid and reliable way?

Q4. Were confounding factors identified?

Q5. Were strategies to deal with confounding factors stated?

Q6. Were the groups/participants free of the outcome at the start of the study (or at the moment of exposure)?

Q7. Were the outcomes measured in a valid and reliable way?

Q8. Was the follow up time reported and sufficient to be long enough for outcomes to occur?

Q9. Was follow up complete, and if not, were the reasons to loss to follow up described and explored?

Q10. Were strategies to address incomplete follow up utilized?

Q11. Was appropriate statistical analysis utilized?

**S3D Table. JBI Critical Appraisal Tool Questionnaire for Cross-Sectional Studies Applied to ZIKV Systematic Review**

| Critical Appraisal Question | Q1 | Q2 | Q3 | Q4 | Q5 | Q6 | Q7 | Q8 | Total QA (%) |
| --- | --- | --- | --- | --- | --- | --- | --- | --- | --- |
| Adams, 2016 | Y | Y | Y | Y | N | N/A | U | N/A | 66.7 |
| Armstrong, 2016 | Y | Y | Y | Y | N | N/A | N/A | N/A | 80 |
| Azeredo, 2018 | Y | Y | Y | Y | Y | Y | Y | Y | 100 |
| Boggild, 2017 | Y | Y | Y | Y | Y | Y | U | Y | 87.5 |
| Brasil, 2016 | Y | Y | Y | Y | N | N/A | N/A | Y | 83.3 |
| Brenciaglia, 2018 | Y | Y | Y | Y | Y | Y | U | Y | 87.5 |
| da Silvo Brito, 2018 | Y | Y | U | U | N | N/A | U | N/A | 33.3 |
| Daudens-Vaysse, 2016 | Y | Y | Y | Y | N | N/A | U | Y | 71.4 |
| Duffy, 2009 | Y | Y | Y | Y | N | N/A | N/A | Y | 83.3 |
| Francis, 2018 | Y | Y | Y | Y | Y | Y | U | Y | 87.5 |
| Hall, 2018 | Y | Y | Y | Y | N | N/A | U | N/A | 66.7 |
| Hamer, 2017 | Y | Y | Y | Y | N | N/A | U | Y | 71.4 |
| Huits, 2019 | Y | Y | Y | Y | N | N/A | Y | Y | 85.7 |
| Jimenez Corona, 2016 | Y | Y | Y | **Y** | N | N/A | N/A | N/A | 80 |
| Journel, 2017 | Y | Y | Y | Y | N | N/A | U | N | 57.1 |
| Lee, 2016 | Y | Y | Y | Y | Y | Y | U | N | 75 |
| Malta, 2017 | Y | Y | Y | Y | Y | Y | Y | Y | 100 |
| McGibbon, 2018 | Y | Y | Y | Y | N | N/A | Y | N/A | 83.3 |
| Mendez, 2017 | Y | Y | Y | Y | N | N/A | U | Y | 71.4 |
| Millet, 2017 | Y | Y | Y | Y | N | N/A | U | Y | 71.4 |
| Parra, 2016 | Y | Y | Y | Y | Y | Y | Y | N/A | 100 |
| Roze, 2017 | Y | Y | Y | Y | Y | Y | Y | Y | 100 |
| Ryan, 2017 | Y | N | Y | Y | N | N/A | N/A | N/A | 60 |
| Schirmer, 2018 | Y | Y | Y | Y | Y | Y | Y | Y | 100 |
| Simon, 2018 | Y | Y | Y | Y | Y | Y | Y | Y | 100 |
| Singapore Zika Study Group, 2017 | Y | Y | Y | Y | N | N/A | U | Y | 71.4 |
| Thomas, 2016 | Y | Y | Y | Y | N | N/A | Y | N/A | 83.3 |
| Vroon, 2017 | Y | Y | Y | Y | Y | Y | Y | Y | 100 |
| Webster-Kerr, 2016 | Y | Y | Y | Y | Y | Y | Y | N/A | 100 |
| Grajales-Muniz, 2019 | Y | Y | Y | Y | Y | Y | Y | Y | 100 |
| Valle, 2018 | Y | Y | Y | Y | Y | Y | Y | Y | 100 |
| Fernandez Martinez, 2019 | Y | Y | Y | Y | Y | Y | U | N/A | 75 |
| Silva, 2019 | Y | Y | Y | N | Y | Y | Y | Y | 87.5 |
| Mercado-Reyes, 2018 | Y | Y | Y | Y | Y | Y | Y | NA | 100 |
| Garcell, 2019 | Y | Y | Y | Y | N | NA | Y | Y | 85.7 |
| Castañeda-Martinez, 2019 | Y | Y | Y | Y | N | NA | Y | NA | 83.3 |
| Sharma, 2019 | Y | Y | Y | Y | N | NA | Y | NA | 83.3 |
| Vasquez, 2019 | Y | Y | Y | Y | Y | Y | Y | Y | 100 |
| Phan, 2019 | Y | Y | Y | Y | N | NA | **Y** | NA | 83.3 |
| Del Carpio-Orantes, 2019 | Y | N | Y | Y | Y | Y | Y | NA | 85.7 |

Q1. Were the criteria for inclusion in the sample clearly defined?

Q2. Were the study subjects and setting described in detail?

Q3. Was the exposure measured in a valid and reliable way?

Q4. Were objective, standard criteria used for measurement of the condition?

Q5. Were confounding factors identified?

Q6. Were strategies to deal with confounding factors stated?

Q7. Were the outcomes measured in a valid and reliable way?

Q8. Was appropriate statistical analysis used?

Note: Causation cannot be inferred from cross-sectional studies, though if no statistical analysis was performed, a point was subtracted from the critical appraisal for the particular study.

Additional notes regarding study classification and critical appraisal:

In classifying study types, we labeled “case series” as those with a particular outcome, such as Guillain-Barré Syndrome (GBS) or other, where authors of the primary study looked back at the exposure, such as ZIKV infection, or if a group with both a particular outcome and exposure were reported on. “Cross-sectional” studies were those that started with a defined population and cases were established by an examination of the population (as opposed to being pre-identified). The reference literature that we used to aid with study classification is cited in our manuscript.

For our critical appraisal, when deciding if studies reported outcomes, such as GBS, in a valid and reliable way, if the authors of the primary article did not describe how they tested for GBS, if confirmed or suspected, and did not relate it to the Brighton Criteria for GBS, then ‘unclear’ was selected on the critical appraisal tool.

When assessing whether primary studies had adequately commented on confounders, we chose ‘yes’ if factors or limitations to the analyses and/or between comparison groups were described by the authors of the primary studies.

As depicted in the Joanna Briggs Institute Critical Appraisal tools, if the primary studies did not include sufficient demographic data of the population or setting such that a reader could extrapolate how it compares to other populations, we answered ‘no’ to these questions.
