# Supplementary material for: Clinical manifestations and health outcomes associated with Zika virus infections in adults: A systematic review
Source: PLoS Negl Trop Dis. 2021 Jul 12;15(7):e0009516. doi: 10.1371/journal.pntd.0009516 (PMC8297931; doi:10.1371/journal.pntd.0009516)
Supplement: S4 Text — (DOCX) [file pntd.0009516.s008.docx]

**S4 Text. Summary of Data Processing for Adult ZIKV Clinical Manifestations and Health Outcomes**

Due to the heterogeneity of ZIKV testing methodologies and reporting of such, we used a methodology outlined below to organize the presentation of health outcomes.

In the Results section of the manuscript, when calculating aggregated numbers of ZIKV clinical outcomes and GBS health outcomes, only studies that met at least 70% of the critical appraisal criteria and reported on ‘confirmed’ ZIKV cases consistent with the WHO ‘confirmed’ ZIKV-case definition were included.

If no definitions were used to categorize cases, then the authors selected the papers and cases that best fit with the WHO definition of ‘confirmed’ to include. The studies in which ‘confirmed’ ZIKV cases by similarity to WHO criteria could be clearly delineated from other subjects in the manuscript were included in Table 2.

The outcomes of GBS cases that correspond to the ZIKV-disease cases resulted in Table 2 are outlined in Table 4 in the manuscript.

The next section of this appendix demonstrates how the remainder of the data from the systematic review that does not meet the above criteria was reported.

If the article delineated the ZIKV case definitions by ‘confirmed,’ ‘probable’ or ‘possible,’ and ‘suspected,’ similar to WHO criteria however the symptomatology or outcomes was not able to be separated between the subjects that had ‘confirmed’ ZIKV infection versus the subjects that had ‘probable,’ ‘possible,’ or ‘suspected,’ then to maximize ability to report, the following rule was applied:

If a cohort of subjects had evidence of ZIKV infection (which may have included RT-PCR, ELISA, or other forms of laboratory testing), then all cases that had evidence of ZIKV infection were detailed to maximize ability to report symptomatology and reported in Table 3 (i.e. when one cannot separate results between ZIKV subgroups, the entire cohort was reported in Table 3).

Similarly, if the ‘confirmed’ cases by the authors’ of the studies definitions were closer to the WHO definition of ‘probable’ or ‘suspected’ then these studies were included in Table 3 in the manuscript.

If the results from a paper could be separated between subjects with ‘confirmed,’ probable,’ or ‘possible,’ and ‘suspected’ ZIKV and could be reported separately, then the symptomatology and outcomes of the ‘confirmed’ subjects were reported in Table 2 and the results pertaining to those with ‘possible,’ ‘probable’ or ‘suspected’ ZIKV were reported in Table 3. Finally, for the studies that had ‘ZIKV-negative’ subjects by laboratory testing but were symptomatic, their symptoms are captured in Table 3 in the manuscript. The outcomes of GBS cases that correspond to ZIKV-disease cases resulted in Table 3 are outlined in Table 5 of the manuscript.

For Tables 4 and 5 in the manuscript, if a paper reported primarily on GBS and symptoms thereof but one could not tease apart which of these symptoms pertained to ZIKV infection, then symptoms were not reported. (For example, this was relevant if a paper had an N = 40 and discussed symptomatology and outcomes of all 40 subjects however did not explicitly delineate which of these outcomes pertained to the subset of subjects that had ZIKV infection). Similarly, if GBS outcomes corresponded to a cohort of GBS patients, not necessarily all pertinent to ZIKV infection, then these studies were not carried forward into Tables 4 and 5 in the manuscript.

This is represented diagrammatically in S2 Fig.
