# Supplementary material for: Clinical manifestations and health outcomes associated with Zika virus infections in adults: A systematic review
Source: PLoS Negl Trop Dis. 2021 Jul 12;15(7):e0009516. doi: 10.1371/journal.pntd.0009516 (PMC8297931; doi:10.1371/journal.pntd.0009516)
Supplement: S5 Text — S5A Table–Clinical and laboratory criteria for Confirmed ZIKV Case by Authors from Primary Articles Included in Table 2 in Manuscript. DENV = Dengue Virus, PAHO = Pan American Health Organization, PRNT = Plaque Reduction Neutralization Test, RT-PCR = Reverse Transcriptase-Polymerase Chain Reaction, RNA = Ribonucleic Acid, VNT = Virus Neutralization Test, WHO = World Health Organization. S5B Table–Case Definitions of ZIKV by Authors of Primary Articles Included in Table 3 in Manuscript. DENV = Dengue Virus, ECDC = European Center for Disease Control Clinical Case Definition = ZIKV infection defined as maculopapular rash with or without fever, and painful joints or muscles or non-purulent conjunctivitis, GBS = Guillain Barré Syndrome, ELISA = Enzyme-Linked Immunosorbent Assay, INS = National Health Institute, PAHO = Pan American Health Organization[45], PCR = polymerase chain reaction, RNA = Ribonucleic Acid, RT-PCR = Reverse Transcriptase-Polymerase Chain Reaction, rRT-PCR = Real-time Reverse Transcriptase-Polymerase Chain Reaction, VNT = Virus Neutralization Test, ZIKV = Zika Virus, ZVD = Zika Virus Disease. (DOCX) [file pntd.0009516.s009.docx]

**S5 Text. ZIKV Case Definitions by Authors of Primary Articles**

This section contains the ZIKV case definitions as specified by the authors of the primary articles included in the systematic review. The definitions for confirmed cases for articles in Table 2 are outlined in S7A. Similarly, Table 3 in the manuscript corresponds to S7B and the case definitions of ZIKV are included in S7B Table.

**S5A Table. Clinical and Laboratory Criteria for Confirmed ZIKV Cases by Authors from Primary Articles Included in Table 2 in Manuscript**

| **Author, Year** | **Clinical case definition** | **Laboratory criteria to confirm ZIKV infection** |
| --- | --- | --- |
| Anaya, 2017[1] | Clinically suspected ZIKV cases reported to Ministry of Health; neurological syndromes with previous probable ZIKV infection (GBS diagnosed based on Asbury/Brighton criteria, encephalitis or transverse myelitis diagnosed by international consortium criteria); cases of previous probable ZIKV infection without neurological syndromes. | RT-PCR, or serum Zika IgM and IgG for neurological cases |
| Cao-Lormeau, 2016[2] | GBS confirmed by neurologist or intensivist based on Brighton criteria. Group 1 controls - hospitalized with non-febrile illness, blood samples taken 7 days post-admission; Group 2 controls - prior confirmed (RT-PCR) ZIKV without neurological outcomes. | Neutralization antibodies, and group 2 confirmed with RT-PCR |
| Geurts vanKessel, 2018[3] | GBS diagnosed according to Brighton criteria (Level 1 or 2 in 92% of subjects) | ZIKV antibodies on NS1 ELISA and if borderline or detectable, confirmed with micro-VNT |
| Chang, 2018[4] | GBS diagnosed according to Brighton criteria (Level 1 or 2) | Reporter Virus Particle (RVP) neutralization assay |
| Lynch, 2019[5] | Clinically diagnosed ZIKV infection; GBS diagnosed by local neurologist (Brighton criteria variably available) | NS1 antibody positivity or a reciprocal 50% neutralization titer against ZIKV strain H/PF/2013 at least two-fold greater than the NT50 titer against dengue virus serotype 2 (DENV2) 16681 |
| Uncini, 2018[6] | ZIKV clinical history of infection; GBS diagnosed according to Brighton criteria (Level 1 30%, Level 2 70%) | Neutralizing antibodies by PRNT90 with a titre of ≥ 128 and IgG antibodies for ZIKV by ELISA and IFI assay |
| Calvet, 2018[7] | PAHO 2016 criteria for suspected case: exanthema and at least 2 of fever, conjunctivitis, arthralgia, myalgia or swollen joints | RT-PCR |
| da Silva, 2017[8] | GBS, transverse myelitis or meningoencephalitis diagnosed according to Brighton criteria or other published guidelines | PAHO and WHO algorithms: RT-PCR in blood or CSF; or positive ZIKV IgM serum or CSF with negative dengue serum serology; or positive ZIKV IgM serum AND CSF with positive dengue serology and dengue CSF IgM negative |
| de Laval, 2018[9] | Cutaneous rash | RT-PCR positive in serum or urine, and negative dengue/chikungunya testing |
| Ng, 2018[10] | fever and maculopapular rash, plus one additional symptom of arthralgia, myalgia, headache or non-purulent conjunctivitis. | RT-PCR |
| Azeredo, 2018[11] | fever, rash during acute phase of infection (up to the 7th day after disease onset) followed by at least two of headache, myalgia or arthralgia, conjunctivitis, pruritus, retro-orbital pain and prostration; OR acute onset of generalized macular or papular rash, pruritus and conjunctival hyperemia | RT-PCR |
| Boggild, 2017[12] | presented to a CanTravNet site after returning from the Americas and physician suspected ZIKV infection | Positive IgM and PRNT at Canada’s National Microbiology Laboratory) or PCR |
| Brasil, 2016[13] | acute onset of generalized macular or papular rash with or without fever | RT-PCR |
| Daudens-Vaysse, 2016[14] | sudden onset of maculopapular rash with or without fever associated with at least two of the three signs conjunctival hyperae-  mia, arthralgia and myalgia, lasting for a week or less and without any other aetiology. | PCR and/or seroneutralization |
| Duffy, 2009[15] | Acute onset of generalized macular or papular rash, arthritis or arthralgia, or non-purulent conjunctivitis | **Confirmed** - Presence of Zika virus RNA or presence of IgM antibody against Zika virus and Zika virus PRNT90 ≥20 and Zika virusPRNT90 : dengue virus PRNT90 ratio ≥4  **Probable case** - Absence of Zika virus RNA and presence of IgM antibody against Zika virus and Zika virusPRNT90 ≥20 and Zika virusPRNT90 : dengue virus PRNT90ratio <4 |
| Francis, 2018[16] | PAHO case definitions - **Prior to April 2016**: fever ≥ 37.2 °C and history of travel to a country endemic for Zika or experiencing a Zika virus outbreak; **after April 2016** - Rash with 2+ of: fever (≥ 38.5°C), conjunctivitis (non-purulent or hyperemic), arthralgia, myalgia, and peri-articular edema, with epidemiological exposure in prior 2 weeks (travel or unprotected sex with traveler) | PAHO case definition - serum RT-PCR positive, or positive Zika IgM and PRNT-90 equal to 20 and four or more times greater than other flaviruses with exclusion of other flaviviruses, or virus detection by molecular methods or immunohistochemistry on autopsy |
| Ho , 2017[17] | fever and rash, with one or more symptoms of headache, arthralgia, myalgia, or conjunctivitis (early in outbreak, also required travel to area with ZIKV transmission) | RT-PCR |
| Huits, 2019[18] | Possible exposure to ZIKV infection within 18 months of consultation - travel to or residence in areas with reported vector-borne transmission of ZIKV restricted to South America and Central America including the Caribbean | ZIKV cases were defined as: Positive or equivocal anti-ZIKV IgM/IgG confirmed with VNT – reciprocal VNT. VNT Ig titer of > 1/10 was positive; travelers with symptoms also tested using RT-PCR |
| Jimenez Corona, 2016[19] | acute fever plus maculopapular rash and conjunctivitis (non-purulent) and, one or more of myalgia, arthralgia, headache or retro-ocular pain, and some epidemiological association | RT-PCR |
| Lee, 2016[20] | Physician suspected ZIKV infection based on symptoms and travel history, or asymptomatic pregnant women with relevant travel history | RT-PCR and ZIKV serology |
| McGibbon, 2018[21] | 2016 CDC/Council of State and Territorial Epidemiologists (CSTE) ZIKV national surveillance case definition | **Confirmed:** either (1) detectable ZIKV RNA or (2) ZIKV IgM-positive with a positive ZIKV PRNT and negative DENV PRNT.  **Probable:** ZIKV IgM-positive with both positive ZIKV andDENV PRNT. |
| Millet, 2017[22] | Clinical criteria as per “National Plan for preparation and response against vector transmitted diseases” (not defined in text, can't locate document) | Case Definition at the Public Health Agency of Barcelona described in “National plan for preparation and response against vector transmitted diseases” - laboratory criteria for confirmed and probable cases |
| Vroon, 2017[23] | temperature instability (< 36 or > 38 °C) with either (i) Suspected ZIKV infection - history and/or clinical presentation with, at least one clinical sign of acute ZIKV infection (i.e., maculopapular rash, myalgia, arthralgia/arthritis, conjunctivitis), or (ii) Suspected ZIKV infection with SIRS criteria, or (iii) SIRS, defined as presentation with at leastone SIRS criterium without history and/or clinical sign of acute ZIKVinfection. | RT-PCR |
| Webster-Kerr, 2016[24] | **May 2015:** acute onset of fever plus any one of generalized macular or papular rash, arthritis or arthralgia, non-purulent conjunctivitis, in someone who resides in or visited epidemic or endemic areas within two weeks prior to onset of symptoms  **Nov 2015:** rash or elevated body temperature (> 37.2 °C) with one or more of (i) arthralgia or myalgia (ii) non-purulent conjunctivitis or conjunctival hyperaemia (iii) headache or malaise”  **Dec 2015**: added neurological manifestations possibly related to ZIKV infection  **2016:** added rash alone for pregnant individuals  GBS meeting Brighton criteria 1-3 or GBS variants | **Confirmed**: RT-PCR.  **Presumptive**: IgM positive for ZIKV and negative for dengue. |
| Gongora_Rivera, 2020[25] | GBS fulfilling level 1 to 3 of Brighton criteria; antecedent ZIKV infection: at least 1 typical ZIKV symptom (rash, joint pain, conjunctivitis) | RT-PCR, or positive ZIKV IgM (variably available) |
| Kozak, 2020[26] | Symptomatic individuals with travel to areas where ZIKV was circulating | RT-PCR |
| Vega, 2018[27] | Acute febrile illness with 2 or more of: exanthema, conjunctivitis, muscle and joint pains, malaise or a headache | RT-PCR |
| Petridou, 2019[28] | Symptoms and/or signs suggestive of ZIKV infection (rash, pruritus, fever, headache, arthralgia or arthritis, myalgia, conjunctivitis, lower back pain, retro-orbital pain) | **Confirmed**: RT-PCR positive  **Seroconversion:** ZIKV IgG negative in an acute blood sample to ZIKV IgG positive in a later blood sample  **Probable**: ZIKV IgM and IgG positive in the earliest blood sample available OR ZIKV IgM strongly positive (normalised optical density ≥2.0) with no follow-up blood sample received but a very compelling clinical presentation; **Likely**: strongly ZIKV IgG positive (normalized optical density ≥2.0) without ZIKV IgM  **Doubtful**: ZIKV IgM positive without ZIKV IgG seroconversion in later samples OR weakly positive ZIKV IgG (normalised optical density < 2.0); Patients who had positive ZIKV serology (either IgM or IgG positive) but had a confirmed or presumptive alternative diagnosis. |
| Hunsberger, 2020[29] | Modified version of PAHO 2016 probable ZIKV infection: any two signs/symptoms (rash, elevated body temperature >37.2 C, arthralgia, myalgia, non-purulent conjunctivitis, conjunctival hyperemia, headache, or malaise) with onset in the previous 7 days preceding the initial visit, not explained by other illness. | Positive RNA |
| Crespillo-Andújar, 2020[30] | Symptoms suggestive of an arboviral infection; and asymptomatic patients who were planning to conceive, pregnant women and their sexual partners returning from a ZIKV endemic area | Positive RT-PCR on any fluid and/or PRNT against ZIKV in the presence of IgM antibodies against ZIKV considered confirmed positive |
| El Sahly, 2019[31] | clinical presentation and history of ZIKV exposure (travel to endemic area or sexual exposure) | RNA in a body fluid specimen or had positive ZIKV IGM and NAb in serum with lower/no NAb against dengue virus 1-4 |
| Grajales-Muniz, 2019[32] | cutaneous exanthema with two or more of fever, headache, conjunctivitis (not purulent/hyperemic), arthralgia, pruritus or retroocular pain and any epidemiological association. | RT-PCR |
| Silva, 2019[33] | Acute febrile illness - measured temperature ≥37.8°C or history of fever in the prior 7 days | RT-PCR |
| Mercado-Reyes, 2019[34] | rash and axillary temperature above 37.2 °C, plus one or more of non-purulent conjunctivitis or conjunctival hyperaemia, arthralgia or myalgias and headache or general malaise *not otherwise explained), and living in a place less than 2200 m above sea level or in a country with confirmed circulation of ZIKV. | Singleplex and multiplex PCR in serum samples or tissue samples |
| Garcell, 2020[35] | WHO definition: rash and/or fever and at  least one ofthe following signs or symptoms: arthralgia or, arthritis  or conjunctivitis (non-purulent/hyperaemic) | RNA in serum or urine |
| Del Carpio-Orantes, 2020[36] | Pruriginous maculopapular exanthema and at least one of fever, cephalea, cunjunctivitis, arthralgia, mialgia, periarticular edema, pruritis, retroorbital pain; plus epidemiological association. | RT-PCR or positive IgM |
| Castañeda-Martinez, 2020[37] | Operational definition for probable or confirmed ZIKV infection according to the Mexican manual of standardized procedures for the epidemiological surveillance of vector-transmitted diseases. (no details in the paper) | RT-PCR |
| Sharma, 2019[38] | Cases of febrile illness with clinical features consistent with Zika virus disease (ZVD); pregnant women; random contacts. | RT-PCR |
| Vazquez, 2019[39] | Acute febrile illness with negative dengue and chikungunya serum PCR | RT-PCR |
| Phan, 2019[40] | **Feb 2016**: fever, rash AND at least one of conjunctivitis, joint pain/muscle pain, or headache, AND travel in/to/from a Zika-affected area within 12 days before symptom onset.  **July 2016**: rash AND at least two of fever more than 38.5 °C, non-purulent conjunctivitis, joint pain/swelling around the joints, or muscle pain. | RT-PCR |

DENV = Dengue Virus, GBS = Guillain-Barre SyndromePAHO = Pan American Health Organization, PRNT = Plaque Reduction Neutralization Test, RT-PCR = Reverse Transcriptase-Polymerase Chain Reaction, RNA = Ribonucleic Acid, VNT = Virus Neutralization Test, WHO = World Health Organization

**S5B Table. Case Definitions of ZIKV by Authors of Primary Articles Included in Table 3 in Manuscript**

| **Author, Year** | **Authors’ Case Definitions of ZIKV** |
| --- | --- |
| Salinas, 2017[41] | Suspected: Rash with ≥ 2 Zika-related symptoms  Probable: Suspected infection plus evidence of recent ZIKV infection (positive or equivocal result for either Zika or dengue virus IgM, Zika virus PRNT90 ≥ 10 and dengue virus PRNT90 < 10) or recent flavivirus (positive or equivocal result for either Zika or dengue virus IgM and Zika and dengue viruses PRNT90 ≥ 10). |
| Styczynski, 2017[42] | Suspected: Rash with ≥ 2 Zika-related symptoms  Authors also reported on recent flavivirus infection (positive or equivocal IgM for ZIKV or DENV) |
| Arias, 2017[43] | Confirmed cases: Instituto Nacional de Salud (INS) criteria for “confirmed” cases which are equivalent to WHO “suspected” cases |
| Baskar, 2018[44] | Recent infection: IgM ZIKV positive |
| Dirlikov, 2018[45] | Evidence of ZIKV infection: RT-PCR or IgM ELISA |
| Van Dyne, 2019[46] | Diagnostic testing by RT-PCR or IgM ELISA |
| Watrin, 2016[47] | Recent history of diarrhea or zika/chikungunya/dengue virus infection. Yap State Department of Health Services and Halstead’s Criteria were utilized to determine which arbovirus on the basis of clinical symptoms and/or laboratory parameters |
| Calvet, 2018[7] | Suspected: PAHO definition of suspected ZIKV cases |
| Lozier, 2018[48] | Current ZIKV infection: ZIKV RT-PCR detected in any specimen  Recent ZIKV infection: ZIKV IgM in absence of DENV IgM  ZIKV positive: Either current or recent ZIKV infection  Recent flavivirus infection: Both anti-ZIKV IgM and anti-DENV IgM without any evidence of of nucleic acid detection |
| Meltzer, 2019[49] | Confirmed infection: Either PCR positive or neutralization assay  Possible infection: ZIKV was positive serology without positive neutralization assay |
| Armstrong, 2016[50] | Testing was performed via RT-PCR or serology  Positive serology testing was represented by anti-ZIKV IgM with ELISA with >= 4 higher neutralization titres compared with that of dengue OR anti-ZIKV IgM with < 4-fold difference in neutralization antibody titres between Zika and dengue and a direct epidemiologic contact that had recent ZIKV infection |
| Brasil, 2016[13] | Confirmed: RT-PCR for confirmed  Suspected: Acute onset of generalized macular or papular rash were suspected |
| Brencialgia, 2018[51] | Confirmed: rRT-PCR  Presumptive: IgM positive only  ZIKV-positive: Confirmed or Presumptive  ZIKV-negative: If negative laboratory evidence but attending physician recorded at least one of the symptoms possible indicators of ZIKV infection |
| Hamer, 2017[52] | U.S. Council of State and Territorial Epidemiologists’ Interim Zika Virus Infection Case Definition, which are separated by:  - Clinical Criteria: Requiring one or more of ZIKV-like symptoms or GBS not precipitated by another etiology  - Probable Case: Meeting clinical criteria with residing in or travel to area with ZIKV spread or having an epidemiologic contact AND either laboratory criteria of ZIKV IgM-positivity AND negative DENV testing AND either no neutralizing testing done or < 4-fold difference in neutralizing titres  - Confirmed Case: Meeting clinical criteria AND laboratory evidence by detection of virus by culture/antigen/RNA or ZIKV IgM with greater than 4-fold neutralizing titres against dengue or other flaviviruses |
| Huits, 2019[18] | This section in Table 3 reports symptoms for ZIKV-negative symptomatic travelers.  ZIKV cases were defined as: Positive or equivocal anti-ZIKV IgM/IgG confirmed with VNT – reciprocal VNT IgG titer of > 1/10 positive; travelers with symptoms also tested using RT-PCR |
| Malta, 2017[53] | Suspected: Hospitalized individuals with neurological manifestations (such as encephalitis, meningoencephalitis, myelitis, or optic neuropathy) or GBS  Probable: Suspected case with history of arbovirus infection (clinical symptoms) in the 60 days before start of neurological symptoms without laboratory testing  Confirmed: Virus isolation |
| McGibbon, 2018[21] | Confirmed: Either detectable RNA or a ZIKV IgM-positive result with a positive ZIKV PRNT and a negative DENV PRNT  Probable: ZIKV IgM positive result with both positive ZIKV PRNT and DENV PRNT |
| Méndez, 2017[54] | ZVD Cases: Symptoms of ZVD with or without lab confirmation.  Laboratory-confirmed ZVD: Presence of clinical symptoms of ZVD and a positive RT-PCR assay |
| Parra, 2016[55] | PAHO case definition |
| Rozé, 2017[56] | Recent infection: RT-PCR ZIKV positive, or ZIKV IgM positive and DENV IgM negative, or ZIKV IgM and DENV IgM positive with positive neutralizing antibodies against ZIKV |
| Schirmer, 2018[57] | Confirmed: RT-PCR positive or ZIKV IgM positive and ZIKV PRNT positive only  Presumed: If ZIKV IgM positive and negative DENV IgM or not tested and a PRNT result positive for ZIKV and DENV IgM |
| Thomas, 2016[58] | Confirmed: Either ZIKV RT-PCR or anti-ZIKV IgM antibody by ELISA with simultaneous negative anti-dengue virus IgM |
| Webster-Kerr, 2017[24] | PAHO case definition |
| Rivera-Correa, 2019[59] | Confirmed: RT-PCR or NT  Probable: IgM and IgM/G  Suspected: IgG |
| Chaumont, 2020[60] | Confirmed: Pathogenic agent or its genome was found inside the CSF  Probable: Agent or its genome was found outside the CSF; or high single IgM measure was found inside the CSF. It was ‘possible’ if a high single IgM measure was found outside the CSF  Suspected: It was of high clinical presumption referred to as clinical cases if in favour of a biologic etiology without proof |
| Lannuzel, 2019[61] | Confirmed: Patients were considered to have confirmed ZIKV recent infection in the presence of (1) detection of viral genome in urine, plasma, or CSF samples or (2) detection of immunoglobulin M (IgM) for ZIKV and plaque reduction neutralization test (PRNT) positive for ZIKV  Probable: Probable recent ZIKV infection in the presence of ZIKV IgM and no dengue IgM  Suspected: Suspected recent ZIKV infection within the previous month of a clinical picture consistent with ZIKV infection that we call typical ZIKV symptoms (rash with 2 or more of the following signs or symptoms: fever, arthralgia, myalgia, conjunctivitis, or edema) |
| Petridou, 2019[28] | Probable: Probable (both IGM/IGG positive/IgM positive no follow up), Likely (IgM negative, IgG positive), Seroconversion |
| Hunsberger, 2020[29] | Suspected: Possible Zika infection |
| Crespillo-Andújar, 2020[30] | Probable: positive IgM ZIKV serology without PRNT confirmation; Past infection positive IgG, negative IgM |
| El Sahly, 2019[31] | Suspected: based on clinical presentation and history of ZIKV exposure (travel to endemic area or exposure) |
| Grajales-Muniz, 2019[32] | Suspected: cutaneous exanthema with two or more of the following: fever, headache, conjunctivitis (not purulent/hyperemic), arthralgia, pruritis, retroocular pain, and any epidemiologic association |
| Valle, 2019[62] | Confirmed: Meets clinical criteria and has detection of ZIKV by RNA and/or Zika virus IgM with neutralizing titres greater than fourfold against dengue or other flavivirus  Probable: Meets clinical criteria and resides in area with ongoing Zika transmission, or epidemiologic link, or association in time and place and IgM with negative dengue and no neutralizing performed, or less than fourfold difference |
| Martinez, 2019[63] | Confirmed: Detection of IgM and positive neutralization or nucleic acid detection or isolation of virus  Probable: Positive serology for IgM, seroconversion of IgG, increase fourfold times of the antibody titre |
| Silva, 2019[33] | Confirmed: RT-PCR |
| Garcell, 2020[35] | Suspected: Rash and/or fever and at least one of the following signs and symptoms: fever, asthenia, anorexia, headache, arthralgia, myalgia, pruritis, rash eye pain conjunctivitis, lymphadenopathy odynophagia, and diarrhea |
| Castañeda-Martinez, 2020[37] | Probable: Operational definition of Zika |
| Vazquez, 2019[39] | Suspected: Acute febrile illnesss |

Of note, PRNT90 is a plaque-reduction neutralization test to detect neutralizing antibodies against a virus. One measures the titer of a subject’s serum required to reduce viral plaques by 90%[64].

DENV = Dengue Virus, ECDC = European Center for Disease Control Clinical Case Definition = ZIKV infection defined as maculopapular rash with or without fever, and painful joints or muscles or non-purulent conjunctivitis, GBS = Guillain Barré Syndrome, ELISA = Enzyme-Linked Immunosorbent Assay, INS = National Health Institute, PAHO = Pan American Health Organization[65], PCR = polymerase chain reaction, RNA = Ribonucleic Acid, RT-PCR = Reverse Transcriptase-Polymerase Chain Reaction, rRT-PCR = Real-time Reverse Transcriptase-Polymerase Chain Reaction, VNT = Virus Neutralization Test, ZIKV = Zika Virus, ZVD = Zika Virus Disease

**References:**

1.         Anaya JM, Rodríguez Y, Monsalve DM, Vega D, Ojeda E, González-Bravo D, et al. A comprehensive analysis and immunobiology of autoimmune neurological syndromes during the Zika virus outbreak in Cúcuta, Colombia. Journal of Autoimmunity. 2017;77: 123–138. doi:10.1016/j.jaut.2016.12.007

2.         Cao-Lormeau VM, Blake A, Mons S, Lastère S, Roche C, Vanhomwegen J, et al. Guillain-Barré Syndrome outbreak associated with Zika virus infection in French Polynesia: A case-control study. The Lancet. 2016;387: 1531–1539. doi:10.1016/S0140-6736(16)00562-6

3.         GeurtsvanKessel CH, Islam Z, Islam MB, Kamga S, Papri N, van de Vijver DAMC, et al. Zika virus and Guillain–Barré syndrome in Bangladesh. Annals of Clinical and Translational Neurology. 2018;5: 606–615. doi:10.1002/acn3.556

4.         Chang AY, Lynch R, Martins K, Encinales L, Cadena Bonfanti A, Pacheco N, et al. Long-term clinical outcomes of Zika-associated Guillain-Barré syndrome. Emerging Microbes and Infections. 2018;7: 4–7. doi:10.1038/s41426-018-0151-9

5.         Lynch RM, Mantus G, Encinales L, Pacheco N, Li G, Porras A, et al. Augmented zika and dengue neutralizing antibodies are associated with guillain-Barré syndrome. Journal of Infectious Diseases. 2019;219: 26–30. doi:10.1093/infdis/jiy466

6.         Uncini A, González-Bravo DC, Acosta-Ampudia YY, Ojeda EC, Rodríguez Y, Monsalve DM, et al. Clinical and nerve conduction features in Guillain−Barré syndrome associated with Zika virus infection in Cúcuta, Colombia. European Journal of Neurology. 2018;25: 644–650. doi:10.1111/ene.13552

7.         Calvet GA, Brasil P, Siqueira AMH, Zogbi HE, Gonçalves BDS, Santos ADS, et al. Zika virus infection and differential diagnosis in a cohort of HIV-infected patients. Journal of Acquired Immune Deficiency Syndromes. 2018;79: 237–243. doi:10.1097/QAI.0000000000001777

8.         Da Silva IRF, Frontera JA, De Filippis AMB, Do Nascimento OJM. Neurologic complications associated with the Zika virus in Brazilian adults. JAMA Neurology. 2017;74: 1190–1198. doi:10.1001/jamaneurol.2017.1703

9.         de Laval F, d’Aubigny H, Mathéus S, Labrousse T, Ensargueix AL, Lorenzi EM, et al. Evolution of symptoms and quality of life during Zika virus infection: A 1-year prospective cohort study. Journal of Clinical Virology. 2018;109: 57–62. doi:10.1016/j.jcv.2018.09.015

10.        Ng DHL, Ho HJ, Chow A, Wong J, Kyaw WM, Tan A, et al. Correlation of clinical illness with viremia in Zika virus disease during an outbreak in Singapore. BMC Infectious Diseases. 2018;18: 1–7. doi:10.1186/s12879-018-3211-9

11.        Azeredo EL, dos Santos FB, Barbosa LS, Souza TMA, Badolato-Corrêa J, Sánchez-Arcila JC, et al. Clinical and Laboratory Profile of Zika and Dengue Infected Patients: Lessons Learned From the Co-circulation of Dengue, Zika and Chikungunya in Brazil. PLoS Currents. 2018;10: ecurrents.outbreaks.0bf6aeb4d30824de63c4d5d745b217. doi:10.1371/currents.outbreaks.0bf6aeb4d30824de63c4d5d745b217f5

12.        Boggild AK, Geduld J, Libman M, Yansouni CP, McCarthy AE, Hajek J, et al. Surveillance report of Zika virus among Canadian travellers returning from the Americas. Cmaj. 2017;189: E334–E340. doi:10.1503/cmaj.161241

13.        Brasil P, Calvet GA, Siqueira AM, Wakimoto M, de Sequeira PC, Nobre A, et al. Zika Virus Outbreak in Rio de Janeiro, Brazil: Clinical Characterization, Epidemiological and Virological Aspects. PLoS Neglected Tropical Diseases. 2016;10: 1–13. doi:10.1371/journal.pntd.0004636

14.        Daudens-Vaysse E, Ledrans M, Gay N, Ardillon V, Cassadou S, Najioullah F, et al. Zika emergence in the French territories of america and description of first confirmed cases of Zika virus infection on Martinique, November 2015 to February 2016. Eurosurveillance. 2016;21: 1–6. doi:10.2807/1560-7917.ES.2015.20.34.30002

15.        Duffy MR, Chen TH, Hancock WT, Powers AM, Kool JL, Lanciotti RS, et al. Zika virus outbreak on Yap Island, Federated States of Micronesia. New England Journal of Medicine. 2009;360: 2536–2543. doi:10.1056/NEJMoa0805715

16.        Francis L, Hunte S-A, Valadere AM, Polson-Edwards K, Asin-Oostburg V, Hospedales CJ. Zika virus outbreak in 19 English- and Dutch-speaking Caribbean countries and territories, 2015-2016. International Journal of Infectious Diseases. 2018;73: 183. doi:10.1016/j.ijid.2018.04.3828

17.        Ho ZJM, Hapuarachchi HC, Barkham T, Chow A, Ng LC, Lee JMV, et al. Outbreak of Zika virus infection in Singapore: an epidemiological, entomological, virological, and clinical analysis. The Lancet Infectious Diseases. 2017;17: 813–821. doi:10.1016/S1473-3099(17)30249-9

18.        Huits R, Maniewski U, Van Den Bossche D, Lotgering E, Tsoumanis A, Cnops L, et al. A cross-sectional analysis of Zika virus infection in symptomatic and asymptomatic non-pregnant travellers: Experience of a European reference center during the outbreak in the Americas. Travel Medicine and Infectious Disease. 2019;27: 107–114. doi:10.1016/j.tmaid.2018.08.007

19.        Jimenez Corona ME, De la Garza Barroso AL, Rodriguez Martínez JC, Luna Guzmán NI, Ruiz Matus C, Díaz Quiñonez JA, et al. Clinical and Epidemiological Characterization of Laboratory-Confirmed Authoctonous Cases of Zika Virus Disease in Mexico. PLoS Currents. 2016;8: ecurrents.outbreaks.a2fe1b3d6d71e24ad2b5afe9828240. doi:10.1371/currents.outbreaks.a2fe1b3d6d71e24ad2b5afe982824053

20.        Lee CT, Vora NM, Bajwa W, Boyd L, Harper S, Kass D, et al. Zika virus surveillance and preparedness-new york city, 2015-2016. Morbidity and Mortality Weekly Report. 2016;65: 629–634.

21.        McGibbon E, Moy M, Vora NM, Dupuis A, Fine A, Kulas K, et al. Epidemiological characteristics and laboratory findings of zika virus cases in New York city, January 1, 2016-June 30, 2017. Vector-Borne and Zoonotic Diseases. 2018;18: 382–389. doi:10.1089/vbz.2017.2223

22.        Millet JP, Montalvo T, Bueno-Marí R, Romero-Tamarit A, Prats-Uribe A, Fernández L, et al. Imported zika virus in a European city: How to prevent local transmission? Frontiers in Microbiology. 2017;8: 1–13. doi:10.3389/fmicb.2017.01319

23.        Vroon P, Roosblad J, Poese F, Wilschut J, Codrington J, Vreden S, et al. Severity of acute Zika virus infection: A prospective emergency room surveillance study during the 2015–2016 outbreak in Suriname. IDCases. 2017;10: 117–121. doi:10.1016/j.idcr.2017.10.007

24.        Webster-Kerr K, Christie C, Grant A, Chin D, Burrowes H, Clarke K, et al. Emergence of Zika Virus Epidemic and the National Response in Jamaica. West Indian Medical Journal. 2017;65: 24–26. doi:10.7727/wimj.2016.488

25.        Gongora-Rivera F, Grijalva I, Infante-Valenzuela A, Camara-Lemarroy C, Garza-Gonzalez E, Paredes-Cruz M, et al. Zika Virus infection and Guillain-Barre syndrome in Northeastern Mexico: A case-control study. PloS one. 2020;15: e0230132. doi:https://dx.doi.org/10.1371/journal.pone.0230132

26.        Kozak RA, Goneau LW, DeLima C, Varsaneux O, Eshaghi A, Kristjanson E, et al. Presence of Flavivirus Antibodies Does Not Lead to a Greater Number of Symptoms in a Small Cohort of Canadian Travelers Infected with Zika Virus. Viruses. 2020;12. doi:https://dx.doi.org/10.3390/v12020140

27.        Vega FLR, Bezerra JMT, Said RF de C, Gama Neto AN da, Cotrim EC, Mendez D, et al. Emergence of chikungunya and Zika in a municipality endemic to dengue, Santa Luzia, MG, Brazil, 2015-2017. Revista da Sociedade Brasileira de Medicina Tropical. 2019;52: e20180347. doi:https://dx.doi.org/10.1590/0037-8682-0347-2018

28.        Petridou C, Simpson A, Charlett A, Lyall H, Dhesi Z, Aarons E. Zika virus infection in travellers returning to the United Kingdom during the period of the outbreak in the Americas (2016-17): A retrospective analysis. Travel Medicine & Infectious Disease. 2019;29: 21–27. doi:10.1016/j.tmaid.2019.03.001

29.        Hunsberger S, Ortega-Villa AM, Powers JH 3rd, Rincon Leon HA, Caballero Sosa S, Ruiz Hernandez E, et al. Patterns of signs, symptoms, and laboratory values associated with Zika, dengue, and undefined acute illnesses in a dengue endemic region: Secondary analysis of a prospective cohort study in southern Mexico. International journal of infectious diseases : IJID : official publication of the International Society for Infectious Diseases. 2020;98: 241–249. doi:https://dx.doi.org/10.1016/j.ijid.2020.06.071

30.        Crespillo-Andujar C, Diaz-Menendez M, Trigo E, Arsuaga M, de la Calle F, Lago M, et al. Characteristics of Zika virus infection among international travelers: A prospective study from a Spanish referral unit. Garcia J Martinez-Sanchez N Rodriguez R Herrero B Lopez F Bartha JL Elorza MD Lafuente MC Hortelano MG M-BE, editor. Travel medicine and infectious disease. 2020;33: 101543. doi:https://dx.doi.org/10.1016/j.tmaid.2019.101543

31.        el Sahly HM, Gorchakov R, Lai L, Natrajan MS, Patel SM, Atmar RL, et al. Clinical, Virologic, and Immunologic Characteristics of Zika Virus Infection in a Cohort of US Patients: Prolonged RNA Detection in Whole Blood. Open forum infectious diseases. 2019;6: ofy352. doi:https://dx.doi.org/10.1093/ofid/ofy352

32.        Grajales-Muniz C, Borja-Aburto VH, Cabrera-Gaytan DA, Rojas-Mendoza T, Arriaga-Nieto L, Vallejos-Paras A. Zika virus: Epidemiological surveillance of the Mexican Institute of Social Security. PloS one. 2019;14: e0212114. doi:https://dx.doi.org/10.1371/journal.pone.0212114

33.        Silva MMO, Tauro LB, Kikuti M, Anjos RO, Santos VC, Goncalves TSF, et al. Concomitant Transmission of Dengue, Chikungunya, and Zika Viruses in Brazil: Clinical and Epidemiological Findings from Surveillance for Acute Febrile Illness. Clinical infectious diseases : an official publication of the Infectious Diseases Society of America. 2019;69: 1353–1359. doi:https://dx.doi.org/10.1093/cid/ciy1083

34.        Mercado-Reyes M, Acosta-Reyes J, Navarro-Lechuga E, Corchuelo S, Rico A, Parra E, et al. Dengue, chikungunya and zika virus coinfection: results of the national surveillance during the zika epidemic in Colombia. Epidemiology & Infection. 2019;147: e77–e77. doi:10.1017/S095026881800359X

35.        Guanche Garcell H, Gutierrez Garcia F, Ramirez Nodal M, Ruiz Lozano A, Perez Diaz CR, Gonzalez Valdes A, et al. Clinical relevance of Zika symptoms in the context of a Zika Dengue epidemic. Journal of infection and public health. 2020;13: 173–176. doi:https://dx.doi.org/10.1016/j.jiph.2019.07.006

36.        del Carpio-Orantes L, Moreno-Aldama N, Sánchez-Díaz J. Clinical characterization of dengue, chikungunya and Zika during 2016 in Veracruz, Mexico. Medicina Interna de Mexico. 2020;36: 147–152.

37.        Castaneda-Martinez F, Valdespino-Padilla M. Characterization of Zika outbreak in rightful owner of IMSS in Lazaro Cardenas, Michoacan, 2016. Medicina Interna de Mexico. 2020;36: 50–58.

38.        Sharma R, Agarwal M, Gupta M, Singh R, Mahavar S, Sharma R, et al. Clinicodemographic profiling of zika outbreak in Jaipur, Rajasthan. Indian Journal of Medical Specialities. 2019;10: 184–189. doi:10.4103/INJMS.INJMS_65_19

39.        Vazquez C, de la Fuente AG, Villalba S, Torales J, Gamarra ML, Ortega MJ, et al. Retrospective detection of Zika virus transmission in Paraguay -- January to December 2016. Weekly Epidemiological Record. 2019;94: 161–165. Available: http://myaccess.library.utoronto.ca/login?url=http://search.ebscohost.com/login.aspx?direct=true&db=rzh&AN=135622635&site=ehost-live

40.        Phan LT, Luong QC, Do THH, Chiu CH, Cao TM, Nguyen TTTTV, et al. Findings and lessons from establishing Zika virus surveillance in southern Viet Nam, 2016. Western Pacific surveillance and response journal : WPSAR. 2019;10: 22–30. doi:https://dx.doi.org/10.5365/wpsar.2018.9.2.014

41.        Salinas JL, Walteros DM, Styczynski A, Garzón F, Quijada H, Bravo E, et al. Zika virus disease-associated Guillain-Barré syndrome—Barranquilla, Colombia 2015–2016. Journal of the Neurological Sciences. 2017;381: 272–277. doi:10.1016/j.jns.2017.09.001

42.        Styczynski AR, Malta JMAS, Krow-Lucal ER, Percio J, Nóbrega ME, Vargas A, et al. Increased rates of Guillain-Barré syndrome associated with Zika virus outbreak in the Salvador metropolitan area, Brazil. PLoS Neglected Tropical Diseases. 2017;11: 1–13. doi:10.1371/journal.pntd.0005869

43.        Arias A, Torres-Tobar L, Hernández G, Paipilla D, Palacios E, Torres Y, et al. Guillain-Barré syndrome in patients with a recent history of Zika in Cúcuta, Colombia: A descriptive case series of 19 patients from December 2015 to March 2016. Journal of Critical Care. 2017;37: 19–23. doi:10.1016/j.jcrc.2016.08.016

44.        Baskar D, Amalnath D, Mandal J, Dhodapkar R, Vanathi K. Antibodies to Zika virus, Campylobacter jejuni and gangliosides in Guillain-Barre syndrome: A prospective single-center study from southern India. Neurology India. 2018;66: 1324–1331. doi:10.4103/0028-3886.241402

45.        Dirlikov E, Major CG, Medina NA, Lugo-Robles R, Matos D, Muñoz-Jordan JL, et al. Clinical features of Guillain-Barré syndrome with vs without zika virus infection, Puerto Rico, 2016. JAMA Neurology. 2018;75: 1089–1097. doi:10.1001/jamaneurol.2018.1058

46.        Van Dyne EA, Neaterour P, Rivera A, Bello-Pagan M, Adams L, Munoz-Jordan J, et al. Incidence and outcome of severe and nonsevere thrombocytopenia associated with zika virus infection-Puerto Rico, 2016. Open Forum Infectious Diseases. 2019;6: 1–9. doi:10.1093/ofid/ofy325

47.        Watrin L, Ghawché F, Larre P, Neau JP, Mathis S, Fournier E. Guillain-Barré Syndrome (42 Cases) Occurring during a Zika Virus Outbreak in French Polynesia. Medicine (United States). 2016;95: 1–8. doi:10.1097/MD.0000000000003257

48.        Lozier MJ, Burke RM, Lopez J, Acevedo V, Amador M, Read JS, et al. Differences in prevalence of symptomatic zika virus infection, by age and sex—puerto rico, 2016. Journal of Infectious Diseases. 2018;217: 1678–1689. doi:10.1093/infdis/jix630

49.        Meltzer E, Lustig Y, Schwartz E. Zika Virus in Israeli travelers: Emergence of Asia as a major source of infection. American Journal of Tropical Medicine and Hygiene. 2019;100: 178–182. doi:10.4269/ajtmh.18-0379

50.        Armstrong P, Hennessey M, Adams M, Cherry C, Chiu S, Harrist A, et al. Armstrong 2016 mm6511e1 Zika travel cases. 2016;65: 286–289.

51.        Brenciaglia M, Noël TP, Fields PJ, Bidaisee S, Myers TE, Nelson WM, et al. Clinical, Serological, and Molecular Observations from a Case Series Study during the Asian Lineage Zika Virus Outbreak in Grenada during 2016. Canadian Journal of Infectious Diseases and Medical Microbiology. 2018;2018: 1-9. doi:10.1155/2018/4635647

52.        Hamer DH, Barbre KA, Chen LH, Grobusch MP, Schlagenhauf P, Goorhuis A, et al. Travel-associated Zika virus disease acquired in the americas through February 2016: A GeoSentinel analysis. Annals of Internal Medicine. 2017;166: 99–108. doi:10.7326/M16-1842

53.        Malta JMAS, Vargas A, Leite PLE, Percio J, Coelho GE, Ferraro AHA, et al. Síndrome de Guillain-Barré e outras manifestações neurológicas possivelmente relacionadas à infecção pelo vírus Zika em municípios da Bahia, 2015. Epidemiologia e servicos de saude : revista do Sistema Unico de Saude do Brasil. 2017;26: 9–18. doi:10.5123/S1679-49742017000100002

54.        Méndez N, Oviedo-Pastrana M, Mattar S, Caicedo-Castro I, Arrieta G. Zika virus disease, microcephaly and Guillain-Barré syndrome in Colombia: Epidemiological situation during 21 months of the Zika virus outbreak, 2015-2017. Archives of Public Health. 2017;75: 1–11. doi:10.1186/s13690-017-0233-5

55.        Parra B, Lizarazo J, Jiménez-Arango JA, Zea-Vera AF, González-Manrique G, Vargas J, et al. Guillain-Barré syndrome associated with Zika virus infection in Colombia. New England Journal of Medicine. 2016;375: 1513–1523. doi:10.1056/NEJMoa1605564

56.        Rozé B, Najioullah F, Fergé JL, Dorléans F, Apetse K, Barnay JL, et al. Guillain-Barré Syndrome Associated with Zika Virus Infection in Martinique in 2016: A Prospective Study. Clinical Infectious Diseases. 2017;65: 1462–1468. doi:10.1093/cid/cix588

57.        Schirmer PL, Wendelboe A, Lucero-Obusan CA, Ryono RA, Winters MA, Oda G, et al. Zika virus infection in the Veterans Health Administration (VHA), 2015-2016. PLoS Neglected Tropical Diseases. 2018;12: 2015–2016. doi:10.1371/journal.pntd.0006416

58.        Thomas DL, Sharp TM, Torres J, Amstrong PA. Local Transmission of Zika Virus — Puerto Rico ,. Morbidity and Mortality Weekly Report. 2016;65: 154–159. doi:10.15585/mmwr.mm6517e2

59.        Rivera-Correa J, de Siqueira IC, Mota S, do Rosario MS, Pereira de Jesus PA, Alcantara LCJ, et al. Anti-ganglioside antibodies in patients with Zika virus infection-associated Guillain-Barre Syndrome in Brazil. PLoS neglected tropical diseases. 2019;13: e0007695. doi:https://dx.doi.org/10.1371/journal.pntd.0007695

60.        Chaumont H, Roze E, Tressieres B, Lazarini F, Lannuzel A. Central nervous system infections in a tropical area: influence of emerging and rare infections. European journal of neurology. 2020;27: 2242–2249. doi:https://dx.doi.org/10.1111/ene.14422

61.        Lannuzel A, Fergé J-L, Lobjois Q, Signate A, Rozé B, Tressières B, et al. Long-term outcome in neuroZika: When biological diagnosis matters. Neurology. 2019;92: e2406–e2420. doi:10.1212/WNL.0000000000007536

62.        Valle J, Eick SM, Fairley JK, Waggoner JJ, Goodman RA, Rosenberg E, et al. Evaluation of Patients for Zika Virus Infection in a Travel Clinic in the Southeast United States, 2016. Southern Medical Journal. 2019;112: 45–51. doi:10.14423/SMJ.0000000000000917

63.        Fernandez Martinez B, Martinez Sanchez E v, Diaz Garcia O, Gomez Barroso D, Sierra Moros MJ, Cano Portero R, et al. Zika virus disease in Spain. Surveillance results and epidemiology on reported cases, 2015-2017. Perez Ruiz M  Cebollada Gracia AD, Vergara Ugarriza A, Huerta Gonzalez I, Grau Sancho P, Cataliba Bosch I, Rojo Moreno ML, Pla Frances A, Blasco de la Fuente A, Javier Viloria Raymundo L, Ruiz Sopena C, Fernandez Arribas S, Peces Jimenez P, Torner Gracia DPE, editor. La enfermedad por virus Zika en Espana Resultados de la vigilancia y epidemiologia de los casos notificados en 2015-2017. 2019;153: 6–12. doi:https://dx.doi.org/10.1016/j.medcli.2018.12.014

64.        Roehrig JT, Hombach J, Barrett ADT. Guidelines for plaque-reduction neutralization testing of human antibodies to dengue viruses. Viral Immunology. 2008;21: 123–32. doi:10.1089/vim.2008.0007

65.        Pan American Health Organization. Zika Resources: Case Definitions. 2016 [cited 23 Aug 2019]. Available: https://www.paho.org/hq/index.php?option=com_content&view=article&id=11117:zika-resources-case-definitions&Itemid=41532&lang=en
