# Supplementary material for: Clinical manifestations and health outcomes associated with Zika virus infections in adults: A systematic review
Source: PLoS Negl Trop Dis. 2021 Jul 12;15(7):e0009516. doi: 10.1371/journal.pntd.0009516 (PMC8297931; doi:10.1371/journal.pntd.0009516)
Supplement: Attachment — Submitted filename: Review Zika Systematic review PNTD.pdf [file pntd.0009516.s010.pdf]

The manuscript by Halani et al is a systematic review of the main clinical manifestations and outcomes observed in adult patients with confirmed of suggested Zika infection. Although this type of study regarding Zika patients is not absolutely new (see two papers in PLoS as examples)

I) Krauer F, Riesen M, Reveiz L, Oladapo OT, Martínez-Vega R, Porgo TV, et al. (2017) Zika Virus Infection as a Cause of Congenital Brain Abnormalities and Guillain–Barré Syndrome: Systematic Review. PLoS Med 14(1): e1002203. https://doi.org/10.1371/journal.pmed.1002203;

II) Leonhard SE, Bresani-Salvi CC, Lyra Batista JD, Cunha S, Jacobs BC, Brito Ferreira ML, et al. (2020) Guillain-Barré syndrome related to Zika virus infection: A systematic review and meta-analysis of the clinical and electrophysiological phenotype. PLoS Negl Trop Dis 14(4): e0008264.)

Our understanding of Zika disease remains incomplete and deserves more investigation.

Minor considerations

1) Page 3, line 62. Since this is the first time the this acronym is showed in your main text, this reviewer recommends to use Zika virus (ZIKV). Thereafter, you should use ZIKV.

2) In this reviewer opinion, Fig 2 may be kept only as a Supplemental file.

3) Page 11, table legend. Did the authors mean El Salvador?

4) In some countries like Brazil, fever was a mandatory symptom to include patients among ZIKV suspected cases at the very beginning of the pandemics. Therefore, the high percentage of fever may be a bias. Did authors analyzed this? Could authors include this in discussion.

5) To improve readability, figure legends should be included at the end of the manuscript, not in the main text.

6) Page 17, lines 257-261. All death cases attributed to ZIKV were laboratory confirmed? Please clarify.

Major considerations

1) Authors stated that they could not perform a meta-analysis given the heterogeneity of the studies, which, unfortunately, weakens the results of the present manuscript. I suggest to address this also in the discussion section, making a comparison to other manuscripts that performed systematic review with meta-analysis for Zika data.

2) According to table 2, in the Anaya study (2017), there were more patients with GBS (29) or neurologic sequel (42) than fever (22) and/or exanthema (25). Similar, for the Cao-Lormeau study, 42 patients had GBS, but 18 of 31 had fever, or 29 of 36 had exanthema.

Please make a comment to clarify this, why more GBS than other more common symptoms.

3) Page 18, lines 263-267. This paragraph reports the results of only one study (Laval et al). To this reviewer it is hard to understand the importance to analyze a potential decrease in quality of life at the third day of infection, which means during acute phase of illness. Since this is not the main goal of this Systematic Review, I suggest removing this paragraph.

4) Page 25, lines 386-389. Authors stated that: "The acute inflammatory demyelinating polyneuropathy subtype (AIDP) subtype of GBS, classically thought to be related to slowed or decreased conduction speed, was more common among ZIKV-infected subjects than the acute motor axonal neuropathy (AMAN) subtype related to the disintegration of neuronal axons [57]".

Similar results were showed by a recent manuscript that was not cited by authors, and should be included into discussion.

: "Leonhard SE, Bresani-Salvi CC, Lyra Batista JD, Cunha S, Jacobs BC, Brito Ferreira ML, et al. (2020) Guillain-Barré syndrome related to Zika virus infection: A systematic review and meta-analysis of the clinical and electrophysiological phenotype. PLoS Negl Trop Dis 14(4): e0008264.

On the other hand, the paper pf Uncini [57] has no data regarding ZIKV.

5) Considering table 4, there are 60 AIDP cases and 57 AMAN, which means there is no statistical support to this statement. But, if we consider Table 5, it seems there is a difference, although it is important to highlight that this table includes the probable/Suspected Cases.
