# Supplementary material for: Clinical manifestations and health outcomes associated with Zika virus infections in adults: A systematic review
Source: PLoS Negl Trop Dis. 2021 Jul 12;15(7):e0009516. doi: 10.1371/journal.pntd.0009516 (PMC8297931; doi:10.1371/journal.pntd.0009516)
Supplement: Attachment — Submitted filename: Halani.docx [file pntd.0009516.s011.docx]

The review by Halani et al summarizes a vast number of manuscripts searchable on databases regarding ZIKV infection and condenses these findings into easy-to-read tables. They have sifted through thousands of manuscripts describing the disease attributed to Zika virus infection. In general, this review is very well done and should be of great interest to a number of people.

Major Concerns

This article is being reviewed in August/September of 2020, but the most recent update to data was performed in December 2018. What is the reason for why a more recent search has not been made to update these findings? It is very likely that most relevant information was reported prior to 2018, but some reports of less likely Zika manifestations might have been reported after 2018. Further, it would help differentiate this review from others to have a more recent dataset.

Minor Concerns

Lines 66-67. Vector-borne infections are from viruses, but “dengue” and “chikungunya” refer here to the disease. Please modify to state “vector-borne diseases.”

Line 62, 71. ZIKV was used before it was defined in line 71. Please define in line 62.

It might read better if paragraphs 2 and 3 in the Introduction were combined.

Lines 161, 162 and several places thereafter. Why are five and eight written out while 11 and 29 numbers? Usually the number name is only written if it begins a sentence. I would recommend listing all in numbers or number names.
