# Supplementary material for: Clinical manifestations and health outcomes associated with Zika virus infections in adults: A systematic review
Source: PLoS Negl Trop Dis. 2021 Jul 12;15(7):e0009516. doi: 10.1371/journal.pntd.0009516 (PMC8297931; doi:10.1371/journal.pntd.0009516)
Supplement: Attachment — Submitted filename: Zika_Adults_Health_Outcomes_SR_ResponsetoReviewers.docx [file pntd.0009516.s012.docx]

Sheliza Halani, BASc, MD

Postgraduate Medical Education

University of Toronto

Phone (416) 357 – 3399

April 12, 2021

Re: PLOS Neglected Tropical Diseases Manuscript PNTD-D-20-01233

Dear Members of the Editorial Committee:

Thank you for your comments and suggestions regarding our manuscript entitled “**Clinical Manifestations and Health Outcomes Associated with Zika Virus Infections in Adults: A Systematic Review**.” We appreciate the opportunity to revise our article.

We have revised the manuscript as suggested and included responses to the comments in the following sections:

1) Reviewer comments within email
2) Reviewer comments in first attachment
3) Reviewer comments in second attachment
4) Additional changes implemented by authors

We believe that we have successfully addressed all comments, and that these revisions have improved the manuscript. We hope that our changes meet with your approval, and would be happy to discuss further as needed.

Thank you again for your consideration. Please do not hesitate to contact us with any questions.

Sincerely,

Sheliza Halani, BASc, MD

Department of Medicine

University of Toronto

On behalf of my co-author Panashe E. Tombindo and the authors of this manuscript.

***REVIEWER COMMENTS WITHIN EMAIL***

**Key Review Criteria Required for Acceptance?**
As you describe the new analyses required for acceptance, please consider the following:

**Methods**
-Are the objectives of the study clearly articulated with a clear testable hypothesis stated?
-Is the study design appropriate to address the stated objectives?
-Is the population clearly described and appropriate for the hypothesis being tested?
-Is the sample size sufficient to ensure adequate power to address the hypothesis being tested?
-Were correct statistical analysis used to support conclusions?
-Are there concerns about ethical or regulatory requirements being met?

**Reviewer #1: A meta-analysis would be of paramount importance in this manuscript, but authors addressed it was not possible due primary studies limitations. Please see my comments in the attached file.**

**Authors’ response:** We have extracted Reviewer #1’s comments from the attachment ‘Review Zika Systematic review PNTD.pdf’ into a subsequent section.

**Reviewer #2: In general, the study designs are appropriate. The only issue is the timing of when the searches stopped. The study objectives are clear and the means taken to eliminate some extraneous data are well-described.**

**Authors’ response:** Thank you for your comments regarding study designs and methods. We have addressed the search date by updating our systematic review in September 2020. The details of this are outlined in subsequent sections of this document and all data updated in the manuscript to reflect the updated search.

**Reviewer #3: The study is aimed at describing the clinical manifestations of ZIKV infection in adults performing a systematic review of observational studies and clinical trials. One of the main challenges described by the authors regards the heterogeneity of such studies, with focus being reported on the diagnostic classification of patients. However, with such a high variation of studies I missed a more comprehensive approach and discussion on the clinical signs and symptoms evaluation as these also can suffer from subjective and diverse methodologies applied. In this aspect I believe the manuscript would benefit of a more comprehensive description on the completeness of information regarding the symptoms presented, intensity and timeline of symptoms emergence.**

**Authors’ response:** From our understanding of the above comment, the reviewer is referring to challenges around obtaining accurate and detailed data about clinical symptoms and signs, such as: relying on self-reporting of symptoms; varying definitions and techniques for measuring signs such as fever; and missing data. We have added a line to the Results section addressing this (pg 20 line 244-245), as well as in the Discussion (pg 29 line 408-412).

**Reviewer #3 continued: One important aspect that was only briefly mentioned by the authors regards the inclusion/eligibility criteria. It should be mentioned that studies using fever and or any symptom as a condition for inclusion will have its estimates biased. It is important to be more descriptive of such symptoms as many guidelines try to differentiate arbovirus infections manifestations based on the prevalence and intensity of such manifestations. ZIKV has been described as causing a high proportion of asymptomatic infections, based largely on retrospective survey data that could be disputed.**

**Authors’ response:** This point is well-taken. The spectrum of ZIKV clinical manifestations is now known to be wider than initially appreciated, and many early case definitions narrow the spectrum by requiring fever and/or rash in the inclusion criteria. For all studies in Table 2 (confirmed ZIKV infection), we have now described the clinical case definitions required for inclusion in Supplementary Table 7A, in addition to the laboratory criteria. In the text, we have added discussion of this issue in Results (pg 12 lines 185-190) and Discussion (pg 28-29 line 402-408).

**Reviewer #3 continued: Regarding the clinical outcomes, mainly regarding neurological manifestations, it is important to mention the study design specifically in the text and tables as this is also influential on how the data is interpreted. The assertion present in the abstract that 6% of cases developed neurologic sequelae is misleading of the real incidence of such complication and must be reviewed.**

**Authors’ response:** We agree that study design is critical in interpretation of data. We have amended Tables 4 and 5 to indicate study design so that this information is easily accessible to the reader.

We have also reworked the text in the Results section on Guillain-Barre Syndrome (pg 21 paragraph starting line 279) to organize studies by design and make this explicit.

We can appreciate that reporting neurologic sequelae at 6% is poorly representative of the true incidence of these rare complications, and that this figure is inflated by case-control studies or case series that focus on neurological sequelae. To address this, we repeated the analysis excluding such studies. Pooling the remaining 13 studies, the incidence of neurological sequelae is 0.3%. We have amended the abstract, author summary, results (pg 20 lines 253-256), and discussion (pg 29 line 415-416) with this more refined analysis.

--------------------

**Results**
-Does the analysis presented match the analysis plan?
- Are the results clearly and completely presented?
-Are the figures (Tables, Images) of sufficient quality for clarity?

**Reviewer #1**: **I pointed some improvements that are necessary to clarify the results. Please see my comments in the attached file.**

**Authors’ response:** We have addressed these below in the last section of this document.

**Reviewer #2**: **Their review of the literature is adequate and appropriate.**

**Reviewer #3**: **There are issues regarding the presentation of the results that are related to the data collected and the limitations of the heterogeneous reporting. Although the authors refrained of performing a meta-analysis, the abstract presents broad frequencies that can be misleading. The review of clinical manifestations has the possibility of providing a broad and comprehensive overview of the clinical picture of this infection, however the detail on the manifestations and the limitations of how the data is reported hamper this contribution that could lay ground for building the need for standardizing the clinical studies data collection and reporting for such studies**

**Authors’ response:** Thank you for your comment. In Tables 2 and 3 we have detailed the symptomatology of clinical infection for subjects with confirmed ZIKV (Table 2) and suspected / probable ZIKV (Table 3). We have represented the frequencies of symptoms in the new Figure 2 (former Figure 3) to surmise the trends of these symptoms with clinical ZIKV infection. If the reviewer would prefer a different format of symptomatology reporting, we would be happy to perform this for a subsequent revision.

--------------------

**Conclusions**
-Are the conclusions supported by the data presented?

-Are the limitations of analysis clearly described?
-Do the authors discuss how these data can be helpful to advance our understanding of the topic under study?
-Is public health relevance addressed?

**Reviewer #1: Yes, authors discussed conclusions and limitations properly.

Reviewer #2: This is a review to their conclusions aren't based upon their own data. However, the discussion does a good job putting their review into context and offers thoughtful caveats and limitations for their interpretations.

Reviewer #3: The authors report some of the main limitations but, in my opinion, fail to highlight the high variability of inclusion criteria, clinical signs and symptoms ascertainment and outcomes measurement.
It is very positive that they shed light on the need to investigate co-infections and describing other needed areas for standardization of the reports.**

**Authors’ response:** We agree with the need to discuss the limitations raised by the reviewer above. We have included discussion of these issues on page 28 paragraph starting line 402.

--------------------

**Editorial and Data Presentation Modifications?**

Use this section for editorial suggestions as well as relatively minor modifications of existing data that would enhance clarity. If the only modifications needed are minor and/or editorial, you may wish to recommend “Minor Revision” or “Accept”.

**Reviewer #1: I addressed some points in my attached review.

Reviewer #2: Overall the data are well summarized. A number of clarifications can be made to enhance the manuscript but are minor. There is one major concern which is addressed elsewhere.

Reviewer #3: (No Response)**

**Authors’ response:** We have addressed these in the next two sections, addressing the major/minor concerns from Reviewer #1 and Reviewer #2.

--------------------

**Summary and General Comments**

Use this section to provide overall comments, discuss strengths/weaknesses of the study, novelty, significance, general execution and scholarship. You may also include additional comments for the author, including concerns about dual publication, research ethics, or publication ethics. If requesting major revision, please articulate the new experiments that are needed.

**Reviewer #1: Please see my comments in the attached file.**

**Response:** We have responded to the reviewer’s comments in the next section.

**Reviewer #2:**

**Major Concerns**

**(1) Comment: This article is being reviewed in August/September of 2020, but the most recent update to data was performed in December 2018. What is the reason for why a more recent search has not been made to update these findings? It is very likely that most relevant information was reported prior to 2018, but some reports of less likely Zika manifestations might have been reported after 2018. Further, it would help differentiate this review from others to have a more recent dataset.**

**Authors’ response:** Our systematic review search was updated using the databases outlined in the Methods section in September 2020.

**Minor Concerns**
 **(2) Comment: Lines 66-67. Vector-borne infections are from viruses, but “dengue” and “chikungunya” refer here to the disease. Please modify to state “vector-borne diseases.”**

**Authors’ response:** We have corrected this statement to vector-borne disease.
 **(3) Comment: Line 62, 71. ZIKV was used before it was defined in line 71. Please define in line 62.**

**Authors’ response:** We have now defined ZIKV in line 61 and removed the definition from line 70.

**(4) Comment: It might read better if paragraphs 2 and 3 in the Introduction were combined.**

**Authors’ response:** We have combined paragraphs 2 and 3 in the Introduction (please see lines 70 to 78).

**(5) Comment: Lines 161, 162 and several places thereafter. Why are five and eight written out while 11 and 29 numbers? Usually the number name is only written if it begins a sentence. I would recommend listing all in numbers or number names.**

**Authors’ response:** We have corrected this so that all are listed in numbers – number names are only used in the updated manuscript if it begins a sentence.

**Reviewer #3: In this study the authors describe the results of a systematic review performed to describe the signs and symptoms of ZIKV infection. This is an important area and topic as there is a broad variation of reports, including the estimates of asymptomatic infections and complications and that ZIKV emergence in Latin America was overlooked for a long period due to overlap of symptoms with other common etiologies of febrile illness. The methods for selecting studies are clear and well applied but some improvement could be done regarding the clinical aspects and reporting of the studies, which could improve the interpretation and relevance of the data.**

**Authors’ response:** Thank for your comment - we agree with this and have included an additional column in Supplementary Table 7 with clinical criteria for inclusion. The first column in Supplementary Table 7 is the clinical case definition for inclusion of subjects in each particular primary article. The second column represents the case definition for confirmed, probable, and suspected cases in each study.

***REVIEWER COMMENTS IN FIRST ATTACHMENT***

**Comment: The manuscript by Halani et al is a systematic review of the main clinical manifestations and outcomes observed in adult patients with confirmed of suggested Zika infection. Although this type of study regarding Zika patients is not absolutely new (see two papers in PLoS as examples)**

**I) Krauer F, Riesen M, Reveiz L, Oladapo OT, Martínez-Vega R, Porgo TV, et al. (2017) Zika Virus Infection as a Cause of Congenital Brain Abnormalities and Guillain–Barré Syndrome: Systematic Review. PLoS Med 14(1): e1002203.** [**https://doi.org/10.1371/journal.pmed.1002203**](https://doi.org/10.1371/journal.pmed.1002203)**;**

**II) Leonhard SE, Bresani-Salvi CC, Lyra Batista JD, Cunha S, Jacobs BC, Brito Ferreira ML, et al. (2020) Guillain-Barré syndrome related to Zika virus infection: A systematic review and meta-analysis of the clinical and electrophysiological phenotype. PLoS Negl Trop Dis 14(4): e0008264.)**

**Our understanding of Zika disease remains incomplete and deserves more investigation.**

**Minor considerations**

**(1) Comment: Page 3, line 62. Since this is the first time the this acronym is showed in your main text,**

**this reviewer recommends to use Zika virus (ZIKV). Thereafter, you should use ZIKV.**

**Authors’ response:** We have corrected this in the text so that it reads ‘Zika virus (ZIKV)’ and thereafter we have used ZIKV.

**(2) Comment In this reviewer opinion, Fig 2 may be kept only as a Supplemental file.**

**Authors’ response:** We have moved Figure 2 to a Supplementary Figure (S4_Fig) to complement the data described in Supplementary File 4. The remainder of the Figures in the text have been renumbered accordingly.

**(3) Comment: Page 11, table legend. Did the authors mean El Salvador?**

**Authors’ response:** We have corrected this to El Salvador in the legend for Table 1.

**(4) Comment: In some countries like Brazil, fever was a mandatory symptom to include patients among ZIKV suspected cases at the very beginning of the pandemics. Therefore, the high percentage of fever may be a bias. Did authors analyzed this? Could authors include this in discussion.**

**Authors’ response:** Please see response to Reviewer #3’s comment in Methods section above. We have addressed this in the discussion (pg 28 paragraph starting line 402).

**(5) Comment: To improve readability, figure legends should be included at the end of the manuscript, not in the main text.**

**Authors’ response:** Thank you for this suggestion for readability. The section on How to Submit Figure Captions on the PLOS Neglected Tropical Diseases website recommended placing figure captions in the manuscript text in read order, immediately following the paragraph where the figure is first cited. We have retained the figure labels in read order, however can move these to the end of the manuscript if requested.

**(6) Comment: Page 17, lines 257-261. All death cases attributed to ZIKV were laboratory confirmed?**

**Please clarify.**

**Authors’ response:** The deaths that are reported in the text are surmised for subjects for whom ZIKV was laboratory-confirmed and among studies that met 70% critical appraisal criteria. Of the fourteen subjects who endured death, the causes of four deaths were shock and coagulopathy in a patient with vascular comorbidity, hospital-acquired pneumonia, cerebral edema and brainstem herniation in a patient with encephalitis, and septic shock. In the study by Mercado-Reyes et al., the fatal cases reported are those with co-infection: histopathology on one subject with CHIKV-ZIKV showed tubule-interstitial nephritis, and changes related to systemic inflammatory response syndrome (SIRS), a second case endured multi-organ failure, and a third the histopathology demonstrated acute demyelinating polyneuropathy, pneumonia, and SIRS findings in the liver and spleen. The cause of death for the remaining seven subjects were not reported in the primary studies.

We have included these details above in lines 268 – 277 pg 21. It is not directly stated that the etiologies of these deaths is ZIKV, especially in the case of co-infections.

***REVIEWER COMMENTS IN SECOND ATTACHMENT***

**Major considerations**

**(1) Comment: Authors stated that they could not perform a meta-analysis given the heterogeneity of the studies, which, unfortunately, weakens the results of the present manuscript. I suggest to address this also in the discussion section, making a comparison to other manuscripts that performed systematic review with meta-analysis for Zika data.**

**Authors’ response:** Our goal for this study was reporting of the breadth of clinical manifestations and commenting on developing on symptomatology complications, hospitalization, ventilation, ICU stay and death. Therefore, given the vast heterogeneity of reporting of these various elements, meta-analysis was not feasible.

We have modified paragraph 2 of the Discussion comparing our study to other systematic reviews and meta-analyses relating to ZIKV and GBS. We moved the comparison to the Barbi et al. (2018) manuscript “Prevalence of Guillain-Barré syndrome among Zika virus infected cases: a systematic review and meta-analysis” to this paragraph, highlighting that among the nine countries in their review, there was a 1.23% estimated prevalence of ZIKV-associated GBS. We have added additional studies from Capasso et al. (2019) and Bautista (2019), additional systematic reviews and meta-analyses on this topic, to compare and contrast our findings regarding the association between GBS and ZIKV.

We have added in citation to the Leonhard, SE et al. (2020) manuscript “Guillain-Barré syndrome related to Zika virus infection: A systematic review and meta-analysis of the clinical and electrophysiological phenotype” in paragraph 1 of the Discussion. We draw upon this reference to demonstrate consistency with our findings regarding electrophysiologic subtype and also regarding admission to ICU (49%) and mortality rate (1%). Leonhard et al. describes these proportions among the category of ‘all cases,’ however we show consistency with our findings regarding 50% ICU admission rate and 3% mortality rate in our subjects with confirmed ZIKV and GBS.

We identified in our study that mortality was uncommon. In paragraph 1 of the discussion, we also compare our results to the mean case fatality rate of ZIKV of 0.02% in the Americas identified in a systematic review by Cardona-Ospina et al. (2019): “Fatal Zika virus infection in the Americas: a systematic review.”

For the purposes of this study as it focuses on adult data, we have not included a comparison to systematic reviews of congenital Zika syndrome or pediatric data. We can include a statement on this if the reviewers feel this would be beneficial to the manuscript.

**(2) Comment: According to table 2, in the Anaya study (2017), there were more patients with GBS (29) or neurologic sequel (42) than fever (22) and/or exanthema (25). Similar, for the Cao-Lormeau study, 42 patients had GBS, but 18 of 31 had fever, or 29 of 36 had exanthema. Please make a comment to clarify this, why more GBS than other more common symptoms.**

**Authors’ response:**

These studies were case-control studies in which cases had GBS and naturally more cases in the studies had GBS, which is why there may be more patients with GBS or neurologic sequelae rather than specific ZIKV-symptoms.

We extracted the data for Table 2 as provided by the authors of the original manuscripts. In the Anaya et al. paper (2017), Figure 5 of their manuscript details the flow diagram of patient recruitment. Through the public health surveillance system, 66 subjects with ZIKV were identified with neurological diseases, and after excluding ‘foreign patients,’ patients with ‘no evidence of neurological compromise,’ patients who ‘declined to participate,’ and patients whose medical records were not available, they identified 42 patients with neurological syndromes for analysis; 29 of which were GBS. Separate from this, they present a table (Table 4) comparing symptoms in patients with ZIKV and GBS to case-controls (ZIKV infection, no GBS). The denominator of cases is 29 in Table 4 of their manuscript and they use this denominator when listing the number of subjects with each symptom (example, fever).

In the Cao-Lormeau study, recruitment was similarly based on identifying subjects with GBS. Forty-two subjects with GBS and ZIKV were identified, with two control groups (control group 1: 98 subjects with a non-febrile illness, and control group 2: acute Zika virus and no neurological symptoms. In Table 1 of the Cao-Lormeau manuscript, they present clinical characteristics of their 42 patients with GBS and ZIKV. In terms of the symptoms of their previous viral illness, the denominators for each symptom vary as presented by the authors. We used the denominators presented in Table 2 of our manuscript. We have added a clarifying statement using two asterisks (**) in the Table 2 footnotes to state that the denominator of symptoms was derived from the original manuscripts.

Additionally, we have on this revision chosen to include the controls from both of these studies in the total number of subjects with confirmed ZIKV for the purpose of enriching the data. Please see section at the bottom of this response document with explanation of the adjustments made.

(3) **Comment:** Page 18, lines 263-267. This paragraph reports the results of only one study (Laval et al). To this reviewer it is hard to understand the importance to analyze a potential decrease in quality of life at the third day of infection, which means during acute phase of illness. Since this is not the main goal of this Systematic Review, I suggest removing this paragraph.

**Authors’ response:** We agree with the reviewer that given that given this paragraph reports the results of one study (Laval et al.), we have removed it from the Results section.

**(4) Comment: Page 25, lines 386-389. Authors stated that: “The acute inflammatory demyelinating polyneuropathy subtype (AIDP) subtype of GBS, classically thought to be related to slowed or decreased conduction speed, was more common among ZIKV-infected subjects than the acute motor axonal neuropathy (AMAN) subtype related to the disintegration of neuronal axons [57]”.**

**Similar results were showed by a recent manuscript that was not cited by authors, and should be included into discussion.**

**Leonhard SE, Bresani-Salvi CC, Lyra Batista JD, Cunha S, Jacobs BC, Brito Ferreira ML, et al. (2020) Guillain-Barré syndrome related to Zika virus infection: A systematic review and meta-analysis of the clinical and electrophysiological phenotype. PLoS Negl Trop Dis 14(4): e0008264.**

**On the other hand, the paper of Uncini [57] has no data regarding ZIKV.**

**Authors’ response:** We have added the Leonhard et al. (2020) manuscript into the discussion to support the data that the AIDP subtype is more common in GBS and ZIKV than AMAN. We have included on line 429 to 431 (pg 29) the following statement: “This is consistent with a meta-analysis of GBS and ZIKV in which the frequency of the AIDP electrophysiologic subtype was 62% followed by 16%[92].”

We have added in a separate reference to support this point from Uncini et al. (2017) “Zika virus infection and Guillain-Barré syndrome: a review focused on clinical and electrophysiologic subtypes.” Although there was some variation in the studies that they review, ultimately the authors concluded that in most cases of Zika infection and GBS, the electrophysiology supported a primary demyelinating neuropathy primarily affecting distal nerve segments.

We have retained the reference from Uncini et al. (2018) “The electrodiagnosis of Guillain-Barré syndrome subtypes: Where do we stand?” as this was the reference used to obtain the definitions of the terms AIDP and AMAN used in the sentence prior.

**(5) Comment: Considering table 4, there are 60 AIDP cases and 57 AMAN, which means there is no statistical support to this statement. But, if we consider Table 5, it seems there is a difference, although it is important to highlight that this table includes the probable/Suspected Cases.**

**Authors’ response:** We agree with the reviewer that it is challenging to draw conclusions from Table 5 as this data is reflective of probable/suspected ZIKV cases. We have added a sentence in the Discussion (now paragraph 2 of Discussion) to illustrate that there is a signal in the data from the probable/suspected ZIKV cases in this study towards the AIDP electrophysiologic subtype being more common than the AMAN, with the caveat that this is to be interpreted in the context of these cases having probable/suspected ZIKV.

In addition, we have included a sentence surmising the total AIDP and AMAN cases in our probable/suspected ZIKV subjects in the Results paragraph that follows Table 5 (see lines 316 to 319 pg 26). “Among the probable and suspected cases of ZIKV with GBS for whom electrophysiologic data was available, 72 of 102 (71%) tested subjects presented with the AIDP subtype, while 13 of 102 (13%) tested subjects exhibited the AMAN subtype. (Table 5)” Of note, the denominators in these calculations is *n=102*. The Arias et al. paper (first row in Table 5), stated that “The conduction velocities of mixed nerves such as the median and ulnar also had impaired sensory latencies, and also the sural nerve was found normal, findings consistent with AMAN subtype neuropathy. In addition, 2 cases (11%) showed unexcitable nerves, and we did not find any abnormality in another 2 patients (tested at days 7 and 11).” Therefore, the authors have amended the first row of Table 5 to state that 0 of 14 subjects tested had the AIDP subtype of GBS.

***ADDITIONAL CHANGES IMPLEMENTED BY AUTHORS***

Additional changes were implemented in the manuscript by the authors which we believe enhance the manuscript quality.

In the Results Section specifically Tables 2 and 3, the authors included controls from the Anaya et al. and Cao-Lormeau et al. studies as the confirmed controls contribute to total confirmed ZIKV subjects in our final numbers. Regarding the Daudens-Vaysse et al. article, initial reporting included ZIKV data from Martinique, the primary territory described in the article, however in this version of the manuscript we revised to include data from all the French Territories of America depicted in the study to add to the dataset. Simon et al. was removed from this study given that despite a study ‘n’ of 15, the total number of subjects with ZIKV was under 10. For consistency with our manuscript’s inclusion criteria, we have removed this study prior to computation of final results.

Figure 2B (formerly Figure 3B) was updated with recomputed numbers of neurological syndromes as per the manuscript.

Given new studies that were added from updating the systematic review, Figure 4 was removed as the data on critical appraisal is depicted in Supplementary File S5.
